# Entropy, Fidelity, and Entanglement During Digitized Adiabatic Quantum Computing to Form a Greenberger–Horne–Zeilinger (GHZ) State

**DOI:** 10.3390/e27090891

**Published:** 2025-08-23

**Authors:** Nathan D. Jansen, Katharine L. C. Hunt

**Affiliations:** Department of Chemistry, Michigan State University, East Lansing, MI 48824, USA

**Keywords:** adiabatic theorem, digitized adiabatic quantum computing, GHZ state, von Neumann entropy, witness operator, entanglement, density matrices

## Abstract

We analyzed the accuracy of digitized adiabatic quantum computing to form the entangled three-qubit Greenberger–Horne–Zeilinger (GHZ) state on two IBM quantum computers and four quantum simulators by comparison with direct calculations using a Python code (version 3.12). We initialized three-qubit systems in the ground state of the Hamiltonian for noninteracting spins in an applied magnetic field in the x direction. We then gradually varied the Hamiltonian to an Ising model form with nearest-neighbor zz spin coupling with an eight-step discretization. The von Neumann entropy provides an indicator of the accuracy of the discretized adiabatic evolution. The von Neumann entropy of the density matrix from the Python code remains very close to zero, while the von Neumann entropy of the density matrices on the quantum computers increases almost linearly with the step number in the process. The GHZ witness operator indicates that the quantum simulators incorporate a GHZ component in part. The states on the two quantum computers acquire partial GHZ character, even though the trace of the product of the GHZ witness operator with the density matrix not only remains positive but also rises monotonically from Step 5 to Step 8. Each of the qubits becomes entangled during the adiabatic evolution in all of the calculations, as shown by the single-qubit reduced density matrices.

## 1. Introduction

Quantum computing has excited considerable interest, because it holds the potential to speed computing processes significantly, enabling calculations that cannot be performed within any realistic time on a classical computer [1,2]. Benioff [3,4,5] and Deutsch [6] suggested the possibility of non-dissipative quantum computers with a lattice of spin-1/2 systems. Fast calculations are made possible by taking advantage of entanglement of qubits whose states are not simply the digital values 0 and 1; instead, the states may be a complex superposition of the ground state |0〉 and the first excited state |1〉 of a two-level quantum system [1,2,3,4,5,6]. The Deutsch-Jozsa theorem provided the first example of an exponential speed-up of quantum computing over classical computing [7,8]. Exponential speed-up has also been proven for the Bernstein-Vazirani algorithm [9] and Simon’s algorithm [10]. Shor’s algorithm speeds the factorization of integers [11]. For simulations of quantum systems with local interactions, Lloyd has shown that the required quantum computer resources do not increase exponentially with system size, as they would on a classical computer [12]. Exponential speed-up has been demonstrated for calculations of thermal rate constants [13], finding eigenvalues and eigenvectors [14], fermionic simulations [15], and transitions in the one-dimensional, nearest-neighbor spin Heisenberg model with disorder [16]. As Richard Feynman remarked, “Nature isn’t classical... and if you want to make a simulation of nature, you’d better make it quantum mechanical, and by golly it’s a wonderful problem, because it doesn’t look so easy” [17].

This work focuses on the production of the entangled three-qubit Greenberger–Horne–Zeilinger (GHZ) state [18,19] by digitized adiabatic quantum computing [20,21] starting from a separable state. The approach relies on the adiabatic theorem of Born and Fock [22]: If a system starts in the ground state of an initial Hamiltonian H_0_, and the Hamiltonian is varied sufficiently slowly to a new form H_f_, the system will remain in the ground state throughout the adiabatic evolution. Rigorous error bounds on the calculated energy were provided first by Kato [23], and then in subsequent developments by others [24,25,26,27,28,29,30]. The error is related to total time taken in the evolution, the norm and derivatives of the Hamiltonian, the spectral gap [23,24,25,26,27,28,29,30], and in some cases, the path length [31,32]. Transitions away from the adiabatic state disrupt the computing process [33,34]. Nenciu [35], and Hagadorn and Joye [36] have derived error bounds on the wave functions obtained by adiabatic quantum computing.

The adiabatic computing process is useful when the ground state of H_0_ is known, and H_f_ is a more complicated Hamiltonian with an unknown ground state [37], so it has applications in quantum chemistry [38,39,40], including calculations of the electronic ground-state energy [38] and the full configuration-interaction wave function for the ground state [39], quantum state preparation with custom gates [41], identification of low-energy conformations of lattice protein models [42], and Berry phase estimation [43]. The approach has also been used to study a chaotic physical system [44] and to find the ground states of frustrated [45] or disordered [46] Ising models. Aharonov et al. have established that adiabatic quantum computation is equivalent to standard circuit-based quantum computation in terms of capabilities and outcomes [47].

Adiabatic quantum computing is also applicable when a problem of a different type can be represented in Hamiltonian form. Examples include digitized adiabatic quantum factorization [48], efficient forms of Grover’s search, interpreted as the search for an item in an unstructured database [49], recall for quantum addressable memory [50], or equivalently identifying the unique input that produces a given output from a black-box Hamiltonian [51]. The method has been applied to the NP-complete problem of finding exact covers; given a set of n bits and a set of 3-bit clauses that require one of the bits to be set to 1 and the other two to be set to 0, an exact cover is a list of triples that satisfy the clauses [52]. The method has also been applied to find cliques in a random graph, i.e., a completely connected sub-graph of an n-vertex graph with a 50/50 chance of a connection between two vertices [53].

Our goals in this work are to determine the effectiveness of the construction of GHZ states by adiabatic quantum computing on IBM’s freely accessible quantum computers and simulators, to find the extent of entanglement, and if possible, to identify a numerical indicator of the relative quality of various computers. While the development of quantum devices has been quite rapid, quantum computers should still be classified as noisy intermediate-scale quantum (NISQ) devices [54,55], a term suggested by John Preskill [54].

We have carried out the adiabatic quantum evolution to form the GHZ state in a direct Python calculation and on the IBM quantum computers ibm_sherbrooke [56] and ibm_brisbane [57]; additionally, we have run the adiabatic quantum evolution on quantum simulators for those computers and simulators for ibm_aachen [58] and ibm_perth [59]. We constructed the simulators for sherbrooke, brisbane, and aachen as described in IBM’s qiskit documentation [60]; this produces noise models based on the individual characteristics of the IBM quantum hardware devices, using current calibration data. For the simulator Fake Perth, the noise model is similar, but the parameters come from data in qiskit Terra [61].

We have used the Trotter–Suzuki formula to carry out the adiabatic quantum computation [62,63]. The discretization process may introduce errors, if the step size is too large. The Python calculation provides a check on the discretization error itself. The remaining errors on the simulators depend on the gate errors, the T_1_ and T_2_ relaxation times, and the readout errors [60]. In addition, runs on the actual quantum computers may be influenced by stray electromagnetic fields.

We have used quantum state tomography [1,64] to determine the density matrices for the 3-qubit systems at each step of the calculations. From the density matrices, we have calculated the von Neumann entropy at each step, in each calculation [65]. We have evaluated the fidelity to a GHZ state using the density matrices [1,66,67]. To probe for GHZ character as the calculation advances, we have used a GHZ witness operator [68,69]. Additionally, we have examined the single-qubit reduced density matrices [70] to test for entanglement [71,72,73,74,75]. For GHZ states of three photons, quantum state tomography has been used in experiments by Resch et al. to reconstruct the polarization state [76].

In Section 2, we describe the digitized adiabatic quantum computing process, the quantum computers and simulators, the quantum circuits, and the quantities used in characterizing the 3-qubit systems in this research. In Section 3 we present the results. The results are analyzed in Section 4, and our principal conclusions are briefly summarized in Section 5. Our results show that the von Neumann entropy remains extremely low throughout the adiabatic quantum computing process in the Python calculations. The three spin-1/2 particles remain in a pure state, and error due to the discretization process appears to be minimal. On the quantum computers ibm_sherbrooke and ibm_brisbane, and on the simulators, the von Neumann entropy increases approximately linearly in the step number during the adiabatic evolution. The single-qubit entropies show that entanglement of all three qubits develops during the computing process in all cases.

## 2. Methods

### 2.1. Digitized Adiabatic Quantum Computing Process

The purpose of adiabatic quantum computing is to find unknown states of the final Hamiltonian. This work focuses on characterizing the quality of the adiabatic quantum computing process on the IBM quantum simulators and the real IBM quantum devices. To test the accuracy of the process, we need to investigate the evolution to a known final state. Therefore, we have used a digitized adiabatic process [20,21] to form the GHZ state, | GHZ 〉 = (1/2)^1/2^ (|000〉 + |111〉). Comparisons with direct construction of the GHZ state are presented with the results in Section 3. We started from the ground eigenstate of three noninteracting spins in a magnetic field B_x_ in the x direction. We have changed the Hamiltonian very gradually from H_0_ = −B_x_ (σ_x,1_ + σ_x,2_ + σ_x,3_) to the final form with zz spin coupling between adjacent qubits, with a coupling constant J_zz_. The final Hamiltonian is given by H_f_ = −J_zz_ (σ_z,1_ ⊗ σ_z,2_ + σ_z,2_ ⊗ σ_z,3_). Here σ_x,n_ and σ_z,n_ are the Pauli spin operators [77] for qubit n and ⊗ denotes the Kronecker product of the operators [78], which is a generalization of the tensor product from vectors to matrices. The time-dependent Hamiltonian has the form H(t) = (t/T) H_f_ + [1 − (t/T)] H_0_, where T is the final, large time. We use an auxiliary variable s defined by s = t/T; s varies from 0 to 1 during the process. For sufficiently slow changes in the Hamiltonian, a system that starts in the ground state of H_0_ at s = 0 arrives at the ground state of H_f_ when s = 1. In the current case, H_f_ has degenerate ground states. In an accurate adiabatic process, the particular ground state in which the system arrives is shown by the density matrix. An alternate approach to generating Schrödinger’s cat states has been used by Bocini and Fagotti, who analyzed the application of a local unitary operator to a quantum transistor chain [79].

### 2.2. Conditions for Validity of the Adiabatic Theorem

A simple “guarantee” of the validity of the adiabatic theorem was analyzed by Albash and Lidar [37]. It depends on the energy gap between states during the adiabatic computing process and the matrix elements of the time-derivative of the Hamiltonian. Here *ε*_0_ denotes the energy of the evolving ground state |*ε*_0_〉, and *ε_m_* is the energy of another state |*ε_m_* 〉. Often, the adiabatic computing process will be successful if

(1) $$\begin{array}{c} \underset{t\in \left[0,T\right]}{max}\frac{\left|\left\langle \varepsilon_{0}\left|\partial_{t}H\right|\varepsilon_{m}\right\rangle \right|}{\left|\varepsilon_{0}-\varepsilon_{m}\right|}\ll 1,\;\forall \;m\neq 0\; \end{array}$$

Albash and Lidar have noted the limitations of this criterion for adiabaticity [37]. Amin [25] has provided an alternative criterion for the applicability of the adiabatic theorem with an interpolated Hamiltonian, *H*(*s*) = A(s) H_0_ + B(s) H_f_ when A(s) is monotonically decreasing and B(s) is monotonically increasing as s increases. This criterion takes the form

(2) $$\begin{array}{c} \frac{1}{T}\underset{s\in \left[0,1\right]}{max}\frac{\left|\left\langle \varepsilon_{0}(s)\left|\partial_{s}H\left(s\right)\right|\varepsilon_{m}(s)\right\rangle \right|}{\left|\varepsilon_{0}(s)-\varepsilon_{m}(s)\right|}\ll 1,\;\forall \;m\neq 0\; \end{array}$$

Specializing to adiabatic evolution of the ground state, Jansen et al. [24] have shown that the necessary run time *T* for an adiabatic quantum computing process scales at worst as 1/Δ^3^, where Δ is the minimum value of the difference between the energies of the ground state and the first excited state. Elgart and Hagedorn have shown that the required run time can be reduced to scale as 1/Δ^2^ (up to a polylogarithmic factor), if the Hamiltonian varies sufficiently smoothly with s [27,37].

### 2.3. Choices of Quantum Computers and Quantum Simulators for Adiabatic Evolution

We have run the digitized adiabatic computing process on two IBM backends, ibm_sherbrooke [56] and ibm_brisbane [57]. We have also run the process on quantum simulators, using the backend noise model simulations for the devices, as described in IBM’s qiskit documentation [60]. For single-qubit gates, the model allows for single-qubit depolarization followed by thermal relaxation. For two-qubit gates, the model accounts for two-qubit depolarizing error followed by thermal relaxation of both qubits. Errors in single-qubit measurements are also included in the models, to allow for the possibility of bit-flip upon read-out [60]. Each model is specific to a particular IBM backend. For each qubit, the model employs T_1_, the decay time for an excited state of a qubit; and T_2_, the decoherence time for an off-diagonal element of the density matrix to fall to (1/e) of its original value [80]. It also employs the gate error for each gate on each qubit, and the gate length of each gate. The noise parameters are obtained from the calibration data for the specific computer being modeled. Thus, the model is a classical simulator of the quantum backend [60]. IBM denotes the models constructed in this way by Fake Sherbrooke, Fake Brisbane, and Fake Aachen. We have also run the adiabatic evolution on Fake Perth, a simulator offered by IBM [61]. The construction of the noise model for Fake Perth is similar to the others, but the parameters come from data in qiskit Terra, rather than from current calibration data. Additionally, we have used classical computing with a Python program (version 3.12) to run the adiabatic process.

### 2.4. Wave Function, Operators, and Circuit for Digitization

The ground eigenstate of H_0_ is separable. Therefore, the qubits should not be entangled when s = 0. We prepared the initial state by applying a Hadamard gate [1,81] to each qubit. The Hadamard gate converts the initial qubit state |0〉 to (1/2)^1/2^ ( |0〉 + |1〉 ). With a Hadamard gate applied to each qubit, we produced a system in the ground state |Ψ_0_ 〉 of the initial Hamiltonian H_0_, with the magnetic field in the x direction. The final wave function is obtained at time T. We determined the value of T and the number of discretization steps N based on numerical experimentation, to ensure that the total evolution time T was large enough to reach the ground state of the final Hamiltonian, while simultaneously ensuring that the step size was small enough for accuracy, but that reductions in the step sizes did not lead to errors due to increased numbers of quantum gate operations. We have tested the accuracy of the discretization procedure by direct classical calculations using Python. Results for 4, 8, and 16 steps are provided in the Supplementary Materials. For this work, we have chosen T = 5 and N = 8. The step times are evenly spaced, from t_1_ = 5/8 to the final value t_N_ = 5, corresponding to 8 values of s = t/T, ranging from s_1_ = 0.125 to s_8_ = 1.

Within each interval, we have approximated the Hamiltonian as a constant operator. Then the wave function can be propagated as [62,63]

| Ψ(s = 1) 〉 = exp[−i H(s_8_) Δt]⋅⋅⋅exp[−i H(s_2_) Δs] exp[−i H(s_1_) Δt] | Ψ_0_ 〉 (3)

with ħ = 1 and Δt = T/N. The Hamiltonian at each step j is

H(s_j_) = −s_j_ J_zz_ (σ_z,1_ ⊗ σ_z,2_ + σ_z,2_ ⊗ σ_z,3_) − (1 − s_j_) B_x_ (σ_x,1_ + σ_x,2_ + σ_x,3_). (4)

The parameters we have used in the Hamiltonian are J_zz_ = 2 and B_x_ = π/2. We have implemented the adiabatic evolution on the quantum computers and quantum simulators via a digitization scheme that includes a first-order Trotterization of the Hamiltonian. This breaks the exponential of the sum of operators into a product of exponentials at each time step: exp[−i H(s_j_) Δt] ≈ exp(−i s_j_ H_f_ Δt) exp[−i (1 − s_j_) H_0_ Δt] [20,21,62,63]. We have constructed the circuit using qiskit, which provides the gates [82]

R_zz_^n,n+1^(s_j_, ϕ) = exp(−i s_j_ ϕ σ_z,n_ ⊗ σ_z,n+1_/2) (5)

and

**Figure 1:**
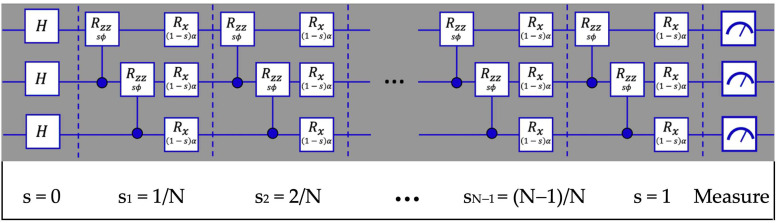
Quantum circuit diagram for discretized adiabatic evolution of a 3-qubit system from the ground state with a magnetic field in the x direction to a GHZ state. The gate structure itself is identical at each step, but the effective rotation angles vary as s increases.

R_x_^n^(s_j_, α) = exp[−i (1 − s_j_) α σ_x,n_/2] (6)

where n and n + 1 are qubit indices, and j is the step index. For the R_zz_ gate we have used ϕ = −2 J_zz_ Δt, and for the R_x_ gate, α = −2 B_x_ Δt. The qiskit circuit is shown in Figure 1.

In Figure 1, the two-qubit rotation gate in Equation (5) is denoted by a box containing R_zz_ above sϕ and the single-qubit gate in Equation (6) is denoted by R_x_ above (1 − s) α to simplify the labels in the diagram. For both gates, s should be replaced by s_j_ at each step j.

### 2.5. Density Matrices and von Neumann Entropy

The density matrix for a three-qubit system is an 8 by 8 matrix, which is determined by coefficients for the basis states |000〉, |001〉, |010〉, |011〉, |100〉, |101〉, |110〉, and |111 〉. The density matrix is Hermitian. Its trace is one, and its eigenvalues are non-negative [83,84]. If the system is in a pure state, then one eigenvalue equals 1 and the other eigenvalues equal 0 [83,84]. In the Python runs, the system remains in a pure state. However, for both the quantum simulators and the real quantum devices, the system is typically in a mixed state. For a mixed state that consists of an ensemble of three-qubit states | Ψ_k_ 〉, each with probability p_k_ of being occupied, the density matrix operator is given by

ρ = Σ_k_ p_k_ | Ψ_k_ 〉 〈 Ψ_k_ |, (7)

where the probabilities p_k_ sum to one [85]. In the Supplementary Materials, we provide explicit expressions for the elements of the density matrix in the basis listed above. The formation of a mixed state results from errors on the NISQ computers or simulators, and from nonadiabatic evolution.

We have determined the density matrices at each step of the discretized adiabatic evolution with the state tomography algorithm in the qiskit_experiments library [86]. The default “maximum likelihood” method was used for fitting to construct the density matrix [87]. The number of measurements needed to find the density matrix varies as 3^n^ in the total number of qubits n, so state tomography is feasible to find the density matrices for nine steps (s_0_ to s_8_) with n = 3. We used 4096 shots per circuit for the tomography. In a measurement on a GHZ state, the outcome should be either |000〉 or |111〉 with a probability of 0.5 for either. With 4096 shots, the random statistical error suggests that the probabilities should lie between 0.484375 and 0.515625. This provides an estimate of the uncertainties in the diagonal elements ρ_000,000_ and ρ_111,111_ of the density matrices. Other errors reflect faults of the NISQ computers. In our calculations, we have retained the figures in the density matrices as provided by the tomography routine in qiskit.

To determine the von Neumann entropy at each step, we have used Mathematica [88] to find the eigenvalues of the density matrix. Then we computed the trace of ρ ln ρ from the scalar product of the vector of eigenvalues with the vector of the natural logarithms of the eigenvalues. The von Neumann entropy is given by [65]

S = − Tr [ρ ln ρ],(8)

up to a factor of the Boltzmann constant k_B_, which is not included here.

### 2.6. GHZ State Fidelity and Purity of the Density Matrix

The fidelity to the GHZ state is f = 〈 GHZ | ρ | GHZ 〉 [1,66]. The fidelity satisfies

〈 Ψ_GHZ_ | ρ | Ψ_GHZ_ 〉 = (1/2) (ρ_000,000_ + ρ_000,111_ + ρ_111,000_ + ρ_111,111_). (9)

That is, the fidelity equals half the sum of the “corner” elements of the 8 by 8 density matrix ρ, indexed by all possible GHZ states with the rightmost index increasing fastest. The density matrix for the GHZ state has entries ρ_000,000_ = ρ_000,111_ = ρ_111,000_ = ρ_111,111_ = 1/2, so the fidelity of the GHZ state itself is 1, as expected. The purity of a state is given by Tr[ρ^2^] [67]; this equals one if the state is pure, and it is less than one if the state is mixed.

### 2.7. GHZ Witness

The three-qubit system starts in a completely separable state, which is the product of three eigenvectors of the Pauli spin matrix σ_x_, one for each qubit. The GHZ state is fully entangled, so it is interesting to watch how entanglement develops during the discretized adiabatic evolution process, and how close the state of the system comes to the GHZ state. Acín et al. showed that the operator W_GHZ_ = (3/4) **I** − P_GHZ_ acts as a witness for the GHZ state [68]. In this expression, **I** denotes the identity operator, and P_GHZ_ is the projection operator for the GHZ state, given by P_GHZ_ = | GHZ 〉〈 GHZ |, with matrix elements

P_GHZ,jk_ = (1/2) (δ_j1_ δ_k1_ + δ_j1_ δ_k8_ + δ_j8_ δ_k1_ + δ_j8_ δ_k8_). (10)

The trace of the product of the witness operator with the density matrix for a coherent state indicates whether the state has a component with GHZ character, as shown by Acín et al. [68]. We note that Eltschka and Siewert have discussed another witness, the tangle [69], which captures the extent of entanglement of three qubits beyond pairwise entanglement [89].

Every three-qubit state can be written in the form

| Ψ 〉 = λ_0_ |000〉 + λ_1_ exp(iθ) |100〉 + λ_2_ |101〉 + λ_3_ |110〉 + λ_4_ |111〉 (11)

by Schmidt decomposition [90], where the coefficients λ_k_ satisfy λ_k_ ≥ 0, the sum of the squares of the λ_k_ values is one, and θ ∈ [0, π]. Thus, five states and six parameters suffice to specify any pure state of three qubits [90]. Acín et al. considered the subset 𝒲 of three-qubit states that contains all states except for the GHZ state [68]. In order to test for GHZ character, it is necessary to determine the maximum possible overlap between the GHZ state and a state in the subset 𝒲.

Because the GHZ state is symmetric with respect to exchange of any of the three qubits, to find the state with maximum overlap, it is sufficient to focus on states in 𝒲 that share the exchange symmetry [68]. Acín et al. note that these states can be written as

| Ψ 〉 = κ_0_ |000〉 + κ_1_ (|100〉 + |010〉 + |001〉 ), (12)

where both κ_0_ and κ_1_ are real, and the state is normalized, so κ_0_^2^ + 3 κ_1_^2^ = 1 [68]. Aside from the GHZ state, the other entangled three-qubit state is the W state [91],

| W 〉 = (1/3)^1/2^ ( |001〉 + |010〉 + |100〉 ). (13)

This state belongs in the subset 𝒲, along with separable states and bipartite entangled states. In looking for overlaps of the GHZ state with the generic state | Ψ 〉 in Equation (12), it is necessary to consider possible rotations of qubits in the wave function | Ψ 〉. The rotations transform |0〉 into α |0〉 + β |1〉 and |1〉 into β* |0〉 − α* |1〉 [68]. The overlap between the GHZ state and a state in the subset 𝒲 is a function of six variables: κ_0_, κ_1_, Re[α], Im[α], Re[β], and Im[β]. Following Acín et al., [68], we have used two Lagrange multipliers η_1_ and η_2_ to maximize the overlap, subject to the constraints κ_0_^2^ + 3 κ_1_^2^ = 1 and |α|^2^ + |β|^2^ = 1. We show details of the analysis. With the auxiliary function g defined by

g(κ_0_, κ_1_, Re[α], Im[α], Re[β], Im[β], η_1_, η_2_)              = κ_0_^2^/2 ( α*^3^ α^3^ + α^3^ β*^3^ + α*^3^ β^3^ + β*^3^ β^3^ )                            + (3/2) κ_0_ κ_1_ ( α*^2^ α^3^ β − α^4^ β*^2^ + α*^2^ β^4^ − α β*^2^ β^3^ )                               + (3/2) κ_0_ κ_1_ ( α*^3^ α^2^ β* − α*^4^ β^2^ + α^2^ β*^4^ − α* β*^3^ β^2^ )                                + (9/2) κ_1_^2^ ( α*^2^ α^2^ β*β − α^3^ β*^3^ − α*^3^ β^3^ + α*α β*^2^ β^2^ )             − η_1_ (κ_0_^2^ + 3 κ_1_^2^ − 1) − η_2_ (|α|^2^ + |β|^2^ − 1), (14)

a necessary condition for the maximum overlap is that the derivatives of g with respect to each of the six variables and with respect to η_1_ and η_2_ all vanish.

We used Mathematica to locate stationary points of the function g. If α and β are restricted to the reals, we find a set of four stationary points with κ_0_ = 0, κ_1_ = ±(1/3)^1/2^, α = ±(1/2)^1/2^, β = −α, η_1_ = −3/4, and η_2_ = −9/4. The overlap | 〈 GHZ | Ψ 〉 |^2^ for these states is 3/4. We find another set of four stationary points with κ_0_ = 1, κ_1_ = 0, α = ±(1/2)^1/2^, β = α, η_1_ = −1/4, and η_2_ = −3/4; these give the overlap | 〈 GHZ | Ψ 〉 |^2^ = 1/4.

If α and β are restricted to be purely imaginary, then we find four stationary points with κ_0_ = 0, κ_1_ = ±(1/3)^1/2^, α = ±(1/2)^1/2^ i, β = −α, η_1_ = −3/4, and η_2_ = −9/4, where the overlap is 3/4. We find an additional set of four stationary points with κ_0_ = 1, κ_1_ = 0, α = ±(1/2)^1/2^ i, β = α, η_1_ = −1/4, and η_2_ = −3/4, which also give an overlap of 1/4.

With α and β both complex, we find a large number of stationary points with κ_0_ = 0, κ_1_ = ±(1/3)^1/2^, η_1_ = −1/18, η_2_ = −1/6, and interrelated values of α and β, all giving an overlap of 1/18. Our results are consistent with the identification of 3/4 as the maximum value of the overlap between the GHZ state and any state in the set 𝒲 [68].

Therefore, the trace of the product of the GHZ witness operator W_GHZ_ = (3/4) **I** − P_GHZ_ with the density matrix ρ for a pure state indicates whether the state incorporates any GHZ character [68]. The result equals 3/4 − f, where f is the fidelity to the GHZ state. If this value is positive, the state has no GHZ character. If it is negative, then the state includes the GHZ state as a component. The result for the GHZ state itself is the minimum possible value, −1/4. We denote the trace of the product of the GHZ witness operator with the density matrix as the “GHZ witness” (as distinct from the GHZ witness operator).

We have evaluated the GHZ witness at each evolution step for the density matrices on ibm_sherbrooke, ibm_brisbane, on four quantum simulators, and in the Python calculation. If the state of the qubits is mixed, the qubit system may have a GHZ component, yet the GHZ witness yields a positive value. Suppose that the system’s density matrix ρ is a linear combination of the GHZ density matrix ρ_GHZ_ and the correct density matrix ρ_0_ at step zero, with ρ = c ρ_GHZ_ + (1 − c) ρ_0_. Then the GHZ witness is positive when c < 2/3, zero when c = 2/3, and negative for c > 2/3. So, the density matrix may incorporate a considerable GHZ component, while the GHZ witness remains positive. In the actual operation of the quantum computers, the states other than GHZ states that are included in the density matrices for the mixed states are not known in general.

A witness operator also exists for the W state in Equation (13); it is (2/3) **I** − P_W_ [68]. If one qubit is measured in the W state, the remaining qubits are still entangled [91].

### 2.8. Entanglement Entropy

We have tested for entanglement by examining the single-qubit reduced density matrices obtained from the full density matrix by taking the trace over the states of the other two qubits. For a pure state, the trace of ρ^2^ is equal to one. For a mixed state, the trace of ρ^2^ will be less than one. For the GHZ state, each of the single qubits is in a mixed state, and the trace of ρ^2^ for each of the single-qubit reduced density matrices is equal to 1/2. The von Neumann entropy for each of the single qubits in the GHZ state is ln 2.

Error correction in the course of adiabatic quantum computing is possible [92], including the use of counterdiabatic methods [93,94,95,96,97], or echo verification [98], but that is beyond the scope of the current work. Similarly, the implementation of methods to speed adiabatic quantum computing [99] is reserved for future work.

## 3. Results

In this section, we provide results we have obtained by quantum state tomography [1,64] for the density matrix, the fidelity to the GHZ state, the von Neumann entropy, the GHZ witness, the purity, and the purity and entropy of the single-qubit density matrices. The results from the Python calculations are given along with the results from ibm_sherbrooke, ibm_brisbane, and the four quantum computer simulators.

### 3.1. Density Matrices from Quantum State Tomography

**Figure 2:**
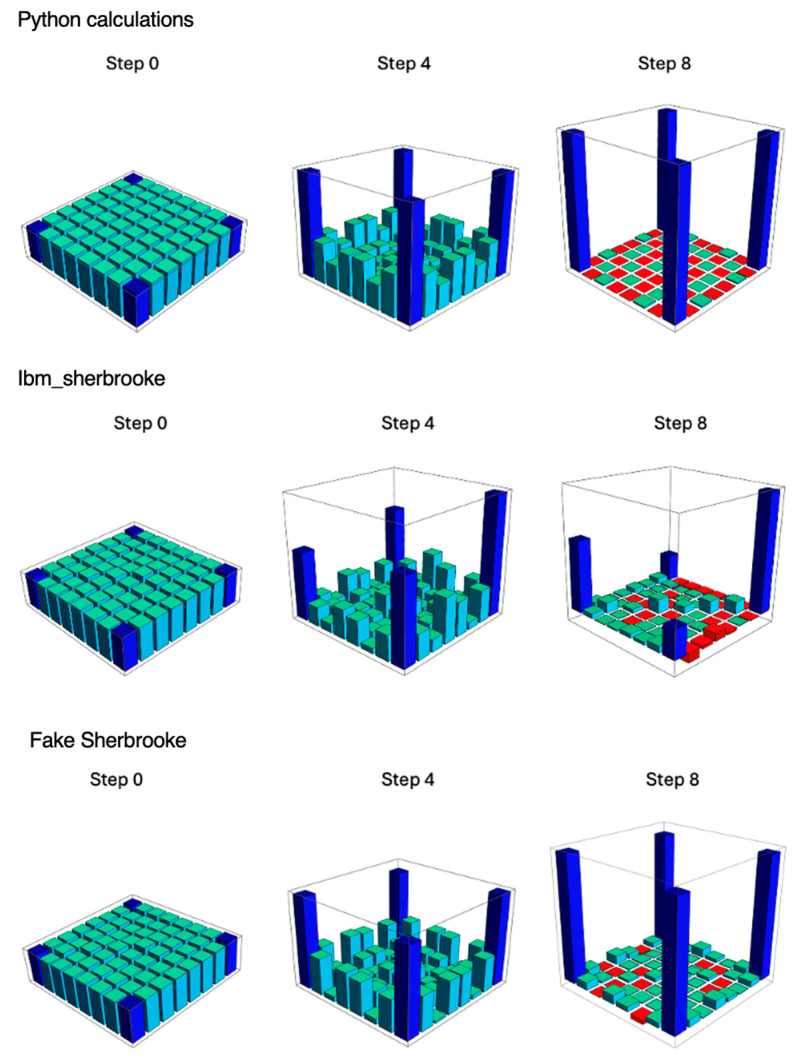
City-scape plots for the density matrices at steps of the adiabatic evolution process, from Python calculations, ibm_sherbrooke and Fake Sherbrooke. Starting at the extreme left and moving toward the lower center of each plot, the positions along the bounding box represent the density-matrix column labels 000, 001, 010, 011, 100, 101, 110, and 111, in order. Starting at the lower center and moving back along the right of the bounding box, the positions represent the row labels in the same order. A city-scape plot is provided in the Supplementary Materials, showing the column and row labels explicitly and marking the blocks that represent the density matrix elements (000,000), (000,111), (111,000) and (111,111). Blue blocks: density matrix elements that are present in the GHZ state; blue-green blocks: other positive matrix elements; red blocks: negative matrix elements.

We have used quantum state tomography to determine the density matrices at steps of the adiabatic evolution process. Figure 2 provides “city scape” plots of the real part of the density matrices. We have selected Step 0, which is the initialization step; Step 4, which is halfway through the calculation; and Step 8, which is the final step, as representative steps for the illustrations. Figure 2 shows the density matrices for the adiabatic evolution process in the Python calculations, on the IBM quantum computer ibm_sherbrooke, and on the classical simulator “Fake Sherbrooke,” which has a noise model based on the characteristics of ibm_sherbrooke. The matrix elements are represented by blocks with heights that are proportional to the value of the real density matrix elements. To clarify the layout of the density matrices in this figure, we have added a diagram to the Supplementary Materials, which shows the column and row labels for the density matrix elements.

Each of the elements of the density matrix should equal 1/8 at Step 0. This condition is guaranteed by the Python code. For both ibm_sherbrooke and Fake Sherbrooke, the state in Step 0 of the adiabatic evolution has been constructed by application of a Hadamard gate to each of three qubits initially in the state |0〉; this produces small faults at Step 0.

The calculated density matrix is Hermitian in all cases, with only very small numerical errors. The density matrix retains four-fold symmetry in the Python calculations. At Step 4, the density matrix on Fake Sherbrooke resembles the density matrix obtained by direct Python calculations. The density matrix element ρ_111,111_ on ibm_sherbrooke is noticeably larger than the other density matrix elements. Our work does not permit us to diagnose the source of this error, but it may result from stray electromagnetic fields, time-correlated noise, crosstalk among qubits on the real quantum computer, or calibration drift. The final density matrix should have four matrix elements with values of 1/2: ρ_000,000_, ρ_000,111_, ρ_111,000_, and ρ_111,111_. All other density matrix elements should vanish. The final density matrix obtained in the Python calculations comes quite close to this form.

### 3.2. The von Neumann Entropy During the Digitized Adiabatic Computing Process

**Figure 3:**
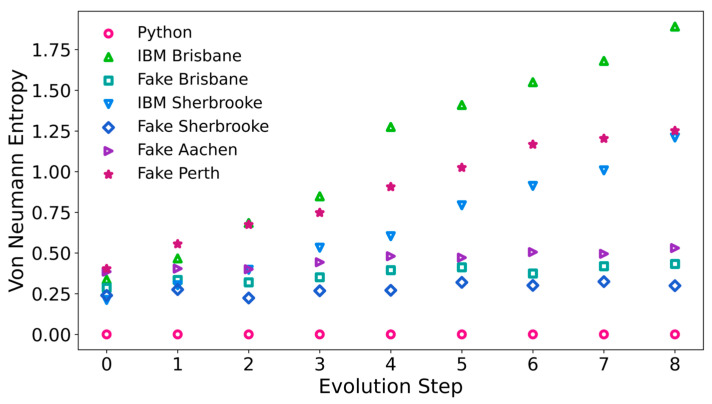
Entropy of the qubits, computed as S = (−1) Tr [ρ ln ρ], plotted as a function of the adiabatic evolution step for two real IBM quantum computers, ibm_brisbane and ibm_sherbrooke, along with four quantum simulators (Fake Brisbane, Fake Sherbrooke, Fake Aachen and Fake Perth), and the results from direct calculations with Python.

We have evaluated the von Neumann entropy of the density matrix as described in the section on methods. The results from the Python calculation, ibm_sherbrooke, ibm_brisbane, Fake Sherbrooke, Fake Brisbane, Fake Aachen and Fake Perth are plotted in Figure 3.

For true adiabatic evolution of a pure state, the von Neumann entropy starts at zero, and remains unchanged throughout the process. We have found small values of the von Neumann entropy in the Python calculation, but they never exceed 1.5 × 10^−14^ at any step. Due to faults on NISQ computers, the evolution process develops nonadiabatic contributions and becomes irreversible. The entropies of the qubits on the IBM quantum computers do not necessarily increase monotonically during the evolution process, because the qubits are not an isolated system. Only the total entropy of the qubits plus surroundings must increase in any irreversible process. In practice, we have found that the von Neumann entropy increases monotonically on ibm_sherbrooke and ibm_brisbane during the evolution process, with near linearity on ibm_sherbrooke. The von Neumann entropy shows an overall tendency to increase on Fake Sherbrooke, Fake Brisbane, Fake Aachen, and Fake Perth, but it is not monotonic in any of these cases. The results from ibm_sherbrooke and ibm_brisbane are rather different from results on the classical simulators for these computers. In the Supplementary Materials, the von Neumann entropies of the three-qubit systems are tabulated at each step of the adiabatic evolution process in the Python calculations, on both quantum computers, and on all four simulators.

The slopes and intercepts of approximate linear fits to the von Neumann entropy as a function of step number are listed in Table 1, along with the fidelity to a GHZ state achieved in the final step. The von Neumann entropy is closest to linearity in terms of the step number for the real IBM quantum computers ibm_sherbrooke and ibm_brisbane, although the R^2^ value on Fake Perth is also high, and it comes very close to the R^2^ value for the real quantum computers. The fit to linearity is weakest for Fake Sherbrooke, but its fidelity to the GHZ state in Step 8 is highest, except for the Python code. The 3-qubit entropy from direct construction of the GHZ state on Fake Perth is 0.686101.

### 3.3. The GHZ Witness During the Digitized Adiabatic Computing Process

**Figure 4:**
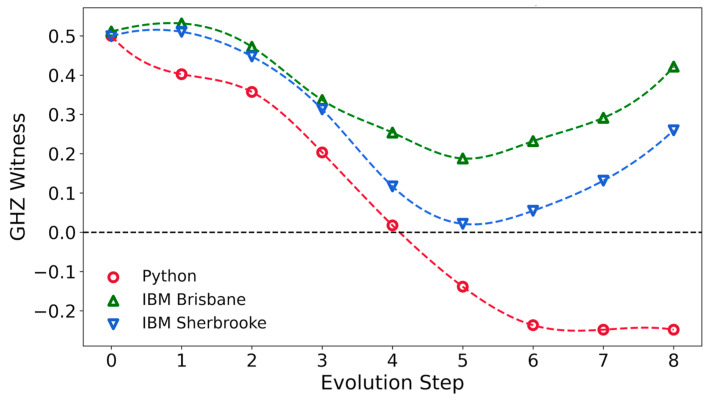
GHZ witness evaluated as a function of the adiabatic computing step. The GHZ witness is negative for a pure state with a GHZ component, but it may be positive for a mixed state with a GHZ component. Dashed lines are added in this figure and following figures as guides for the eyes.

Figure 4 shows the GHZ witness as a function of step number for the Python code, and the quantum computers ibm_brisbane, and ibm_sherbrooke. For the Python code, the GHZ witness becomes negative at Step 5, and for Steps 6, 7, and 8, its value is close to the result of −1/4 for the true GHZ state. At Step 8 (the final step), the GHZ witness obtained with the Python code is −0.24799.

In runs on ibm_brisbane and ibm_sherbrooke, the GHZ witness is always positive. The minimum value of the witness occurs in Step 5 for these computers. The density matrices on these computers may still incorporate considerable GHZ character, because their states are mixed.

**Figure 5:**
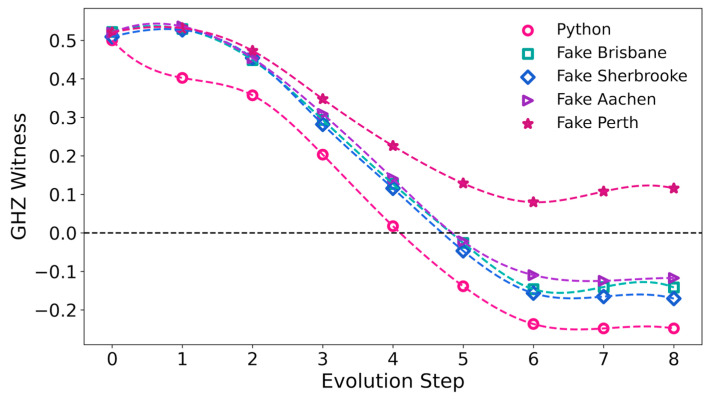
GHZ witness evaluated as a function of the discretized adiabatic quantum computing step for the Python calculations and the quantum simulators Fake Perth, Fake Aachen, Fake Brisbane, and Fake Sherbrooke. The density matrices from Python calculations and from the simulators clearly incorporate a GHZ component, except for Fake Perth.

Correspondingly, Figure 5 shows the GHZ witness for the Python code and for the quantum simulators. As we found for the Python code, the GHZ witness is negative in Steps 5 through 8 on Fake Sherbrooke, Fake Brisbane, and Fake Aachen, with witness values of −0.170438, −0.141524, and −0.117094 in Step 8, respectively. The GHZ witness on the Perth simulator remains positive throughout the adiabatic evolution process with a minimum of 0.079753, which occurs at Step 6.

The GHZ witness for the direct construction of the GHZ state on Fake Perth is −0.083899. In the Supplementary Materials, the GHZ witness values for the 3-qubit states are tabulated at each step of the adiabatic evolution process in the Python calculations, on both quantum computers, and on all four simulators.

### 3.4. Fidelity to the GHZ State

We have not included a plot for the fidelity to the GHZ state in the main text, because the fidelity f is directly related to the GHZ witness g by f = 3/4 − g. The fidelity is tabulated and plotted for the various runs in the Supplementary Materials. It is useful to consider a few numerical values of the fidelity. The fidelity to the GHZ state at the final step of the Python calculations is 0.99799. Cutting the step size Δs in half increases that fidelity slightly, to 0.99811. This indicates that the 8-step discretization is not a substantial source of error in itself. On ibm_sherbrooke, the fidelity to the GHZ state reaches its maximum at Step 5, where it is 0.728431. Then the fidelity drops monotonically in Steps 6, 7, and 8; at the final step, the fidelity is 0.490356. Results obtained with Fake Sherbrooke are more accurate, with a fidelity of 0.920438 achieved at the final step. The fidelity obtained in the direct construction of the GHZ state on Fake Perth is 0.833899.

### 3.5. Purity as an Indicator That States Are Mixed

We have also determined the purity of the three-qubit density matrices, given by Tr[ρ^2^] [67] during the adiabatic quantum computing process. This provides an indicator of the extent of mixing. In the current case, for an ideal adiabatic process, the purity should start at 1 and remain there throughout. On ibm_sherbrooke, the purity drops from 0.919792 at Step 0 to 0.471861 at Step 8, while on ibm_brisbane, the purity drops from 0.856765 at Step 0 to 0.184950 at Step 8. The fall-off of the purity is nearly linear. On ibm_sherbrooke, the intercept of the purity versus step number is 0.94230, the slope is −0.05510, and the R^2^ value is 0.98601. On ibm_brisbane, the intercept is 0.86303, the slope is −0.08794, and the R^2^ value is 0.97974. The differences in the characteristics of ibm_sherbrooke [56] and ibm_brisbane [57], in terms of the median T_1_ and T_2_ values for the qubits, and the median ECR, SX, and readout errors [60], became evident during adiabatic quantum computing to produce the GHZ state. The purity obtained in the direct construction of the GHZ state on Fake Perth is 0.704458.

The imaginary parts of the density matrices are small, but they may not be negligible. For example, at Step 8 of the adiabatic computing process using Python, the purity of the system calculated with the full density matrix is 1.000000, while the trace of the square of the real part of the density matrix is 0.996316. If the step size is cut in half, at the final step in the Python calculations (with version 3.12), the trace of the square of the real part of the density matrix (taken by itself) is 1.000000. The purities of the three-qubit density matrices are tabulated in the Supplementary Materials for the runs on ibm_sherbrooke and ibm_brisbane, and for the Python calculations.

**Figure 6:**
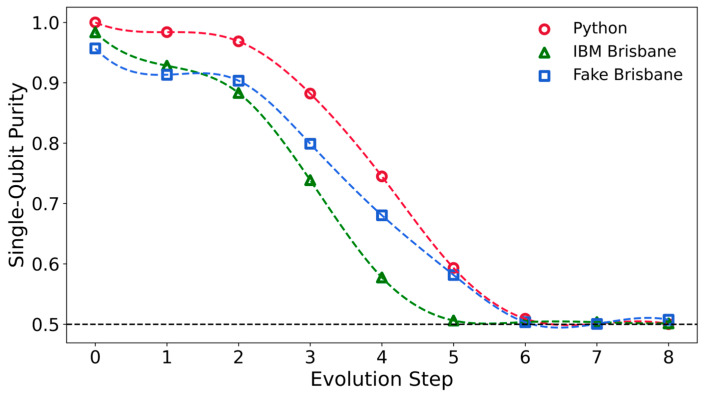
Purity of qubit q[0] as a function of the discretized adiabatic quantum computing step for the Python calculation, ibm_brisbane, and the Brisbane simulator.

We have also determined the purity of the reduced density matrices for each of the three qubits during the adiabatic evolution process in the Python calculations, on ibm_brisbane, ibm_sherbrooke, and their simulators. Figure 6 shows the purity of qubit q[0], computed as Tr[ρ_0_^2^], at each step in the Python code, on ibm_brisbane and on the brisbane simulator. Here, ρ_0_ is the reduced density matrix for qubit q[0], obtained by taking the trace over states of the other two qubits. The purity is 1 for a pure state; it should be 0.5 for single-qubit mixed states derived from the GHZ state. The reduced density matrices are expected to show that the individual qubits are in mixed states, even though the three-qubit state remains pure in the Python calculations. In all cases, the purity drops swiftly between Steps 2 and 5, though it drops more slowly in the Python calculations than on ibm_brisbane. Qubits q[0] and q[2] have identical purities at each step in the Python code, but the purity of q[1] differs.

At Step 8, the purity of the density matrix on Python is 0.500036 for q[0] and q[2], and 0.500309 for q[1]. On both real quantum computers and both of their simulators, the purities of the three qubits differ. For example, on Fake Sherbrooke, the purity at Step 8 is 0.506693 for q[0], 0.500079 for q[1], and 0.505616 for q[2].

### 3.6. Single-Qubit von Neumann Entropies

**Figure 7:**
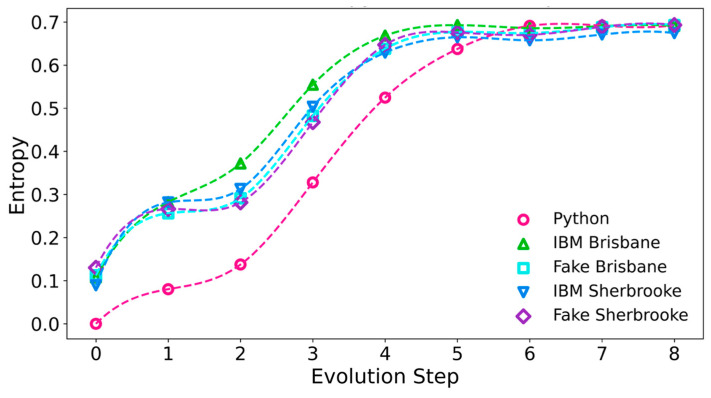
Single-qubit entropies for qubit q[1] during the adiabatic evolution. An entropy of ln 2 (≈0.693147) applies for a completely mixed state and shows that the qubit is entangled.

We have determined the von Neumann entropies of the individual qubits at each step in the adiabatic evolution. We computed the single-qubit entropies using the equation S_n_ = (−1) Tr [ρ_n_ ln ρ_n_], in terms of the reduced density matrix for the individual qubit n. In the Python calculations, the entropy of each qubit at Step 0 is zero, indicating that each starts in a pure state, as expected. In the runs on ibm_sherbrooke, ibm_brisbane and their quantum simulators, we have found a non-zero entropy at Step 0, showing that the state of each qubit is already mixed at the start of the process; typically, the entropy at Step 0 is close to 0.1. The entropy of each of the qubits remains larger than the entropy in the Python calculations until Step 6, when the entropy has almost reached the final value of ln 2 for a completely mixed state. For ibm_sherbrooke, Fake Sherbrooke, and Fake Brisbane, the plots of the entropy versus the adiabatic computing step are quite similar. They show an initial shoulder, followed by a rapid rise, coming close to 0.6 by Step 5, and then close to ln 2 in Steps 6, 7, and 8. On ibm_brisbane, the pattern was similar for q[1], but for q[0] and q[2], ibm_brisbane was an outlier at the time of our runs, with an especially high entropy for q[2] at all steps. Figure 7 shows the von Neumann entropies of q[1] for various runs. For the quantum computers and quantum simulators, these curves are the most similar and the smoothest.

The single-qubit entropy of q[1] from a direct construction of the GHZ state on Fake Perth is 0.691898 and its purity is 0.503743. The results for the purities and the entropies of the single qubits show that they are entangled in all cases, even though the GHZ witness remains positive on ibm_sherbrooke and ibm_brisbane throughout the computing process. This is possible because the three qubits are in mixed states.

The single-qubit purities and the single-qubit entropies are tabulated in the Supplementary Materials at each step of the discretized adiabatic quantum computing process for the runs on ibm_sherbrooke, ibm_brisbane, Fake Sherbrooke, Fake Brisbane, and the Python calculations. In the Supplementary Materials we also provide plots of the single-qubit purities and the single-qubit entropies for qubits q[0] and q[2] in each of these cases.

## 4. Discussion

The GHZ witness operator creates a sharp division between coherent states that include GHZ character and those that do not. The highest possible value of the overlap between a GHZ state and a non-GHZ state | Ψ 〉 is 3/4, as given by | 〈 GHZ | Ψ 〉 |^2^. Hence, the witness operator W_GHZ_ = 3/4 **I** − | GHZ 〉 〈 GHZ | serves to detect GHZ character in a wave function [68]. If the trace of the product of the density matrix with the GHZ witness operator is negative for a coherent state, then the density matrix includes a GHZ component; otherwise, it does not. In this work, we found that the GHZ witness in the final step of the Python calculations was −0.247990, very close to the value of −1/4 for a pure GHZ state.

If the system’s state is mixed, the density matrix may contain an appreciable GHZ character, even though the GHZ witness remains positive. For example, for a three-qubit system with a density matrix that is a linear combination of a GHZ density matrix and the density matrix for the separable initial state that we have constructed, the GHZ coefficient must exceed 2/3 before the trace of the density matrix with the GHZ witness operator becomes negative. The density matrix is mixed on ibm_sherbrooke and ibm_brisbane, and the GHZ witness never becomes negative for the evolution on these computers, even though all three qubits become entangled. The quantum simulators are also in mixed states, but with larger GHZ components. On the quantum simulators modeled after ibm_sherbrooke, ibm_brisbane, and ibm_aachen, the GHZ witness became negative by Step 5 of the eight-step adiabatic computing process used in this work, as it did in the Python calculations. The values of the GHZ witness at Step 8 ranged between −0.170438 and −0.117094 on the simulators.

To test for entanglement of the qubits, we determined the purity of the single-qubit reduced density matrices and the single-qubit entropies. For both quantum computers, ibm_sherbrooke and ibm_brisbane and their quantum simulators that we examined, the purity of each qubit’s state started close to 1 at Step 0, and then dropped close to 0.5 at Steps 6, 7, and 8. The value of 1 indicates that the qubit state is separable, as expected; the value of 0.5 is characteristic of a fully mixed state. The entropy of qubit q[1] (the “middle” qubit) on the quantum computers and simulators starts close to 0.1, rather than 0; it increases and approaches ln 2 in later steps of the evolution. The qubit entropy always rises faster for the quantum computers and their simulators than in the Python calculations, reaching a value very close to ln 2 by the final step in all cases. The entropy of qubit q[2] rises especially quickly on ibm_brisbane. Both the purities and the entropies of the single qubits indicate that they are entangled.

Entanglement of the qubits is key to gaining quantum advantage [100]. In future work, we plan to examine the preparation of entangled states of larger numbers of qubits, as in the 2017 work of Song et al. with ten-qubit entanglement [101], the 2020 work of Sager et al. demonstrating entanglement in the formation of an exciton condensate of photons on a 53-qubit quantum computer [102], and the 2025 work of Liao et al. with 78-qubit entanglement and quantum error correction [103]. Tabia et al. have addressed the issue of transitivity of entanglement, for an arbitrarily large number of qubits [104]. We also plan to investigate the loss of entanglement after it has formed. Fröwis et al. have discussed the extension of “quantumness” to the macroscopic scale; they have noted that the quantum characteristics of macroscopic quantum systems are fragile to noise-induced decay [105].

Quantum state tomography to determine the density matrices will become impracticable as the total number of qubits n increases, due to its scaling as 3^n^ [1]. However, the states of larger numbers of qubits can still be investigated using the Shannon entropy [106]. Jansen et al. have found that the Shannon entropy of measurement outcomes on n-qubit Schrödinger’s cat states is nearly linear as a function of n, in work with up to 15 qubits [107]. The slope of the Shannon entropy of n-qubit cat states versus n provides an indicator of the accuracy of current NISQ computers [107]. In future work, it may be possible to take advantage of recently announced quantum computers with high fault-tolerance [108,109].

We also plan to investigate quantum thermodynamic effects in qubit systems. Kosloff has shown that the Laws of Thermodynamics derive from quantum principles [110]. Dann and Kosloff have separated energy changes on the quantum scale into heat and work [111]. They have also extended quantum thermodynamic analyses to open systems [112,113], with results that are relevant to the operations of qubits in quantum computers. Kosloff and coworkers have analyzed the operation of quantum heat engines and quantum refrigerators [114,115], identifying a kind of friction at the quantum level, based on nonadiabaticity, dissipation, and decoherence [115]. They have also examined thermodynamic limits in finite-time quantum operations [116,117]. In fact, quantum engines have been constructed experimentally. For example, Roßnagel et al. have determined the power and efficiency of a quantum engine consisting of a single ion in a Paul trap with a tapered geometry, which is coupled to hot and cold reservoirs in alternation [118]. Klatzow et al. have demonstrated efficiency beyond the Carnot limit in experimental studies of quantum heat engines that involve states of nitrogen vacancy centers in diamond lattices [119]. We are interested in evaluating entropy changes that accumulate during a cycle in a quantum engine. For ideal operation, there would be no net change in the entropy of the working material during the cycle, but the quantum operation may be non-ideal. This work is expected to become increasingly important with the growing emphasis on the production of devices on the quantum scale [120].

## 5. Conclusions

The slope and intercept of the plot of the von Neumann entropy versus adiabatic computing step in the formation of the GHZ state indicate the accuracy of the quantum computer at the time of the run. The results may also be useful in monitoring the performance over time after calibration runs. We show that the qubits become entangled during the evolution process, even though the GHZ witness may remain positive, because the state of the system is mixed.

## Figures and Tables

**Table 1:** Best linear fits to the von Neumann entropy as a function of the step number, the R^2^ values for the fits, and the fidelity to the GHZ state in the final step.

Computer	Slope	Intercept	R^2^ of Fit	Monotonic	Fidelity
ibm_sherbrooke	0.123385	0.169847	0.989866	Yes	0.490356
ibm_brisbane	0.202520	0.316726	0.982477	Yes	0.328328
Fake Sherbrooke	0.009875	0.240661	0.622159	No	0.920438
Fake Brisbane	0.016841	0.302194	0.853455	No	0.891524
Fake Aachen	0.018197	0.384661	0.933290	No	0.867094
Fake Perth	0.109992	0.441399	0.982121	No	0.634522

## Data Availability

Data supporting the conclusions of this article are included in the article and in the Supplementary Materials. The density matrices will be made available by the authors on request.

## Supplementary Materials

The following supporting information can be downloaded at: https://www.mdpi.com/article/10.3390/e27090891/s1, Table S1: Von Neumann entropies of the three qubits. Table S2: Fidelities to the GHZ state. Table S3: GHZ witness values for both quantum computers, all four quantum simulators, and the Python calculations. Table S4: Single-qubit purities, and Table S5: Single-qubit entropies at each step in the evolution process, for both quantum computers, their simulators, and the Python calculations. Table S6: Purities of the three-qubit density matrices at each step in the evolution process on ibm_sherbrooke, ibm_brisbane, and in the Python calculations. Table S7: GHZ witness from Python calculations with 4, 8, and 16-digitzation steps. Table S8: Fidelity to the GHZ state in 4-, 8-, and 16-step adiabatic evolution processes. Table S9: Single-qubit entropies of q[1] in Python calculations with 4, 8, and 16 steps, and Table S10: Single-qubit purities of q[1] in Python calculations with 4, 8, and 16 steps. Figure S1: Labeled city-scape plot. Figure S2: Fidelities to the GHZ state. Figure S3: Single-qubit purities. Figure S4: Single-qubit entropies. Figure S5: Purities of the three-qubit states on the two quantum computers and in the Python calculations. Figures S6 and S7: GHZ witness and fidelity to the GHZ state in Python calculations with 4, 8, and 16 steps. Figures S8 and S9: Single-qubit entropies and single-qubit purities for q[1] in Python calculations with 4, 8, and 16 steps. Commentary on density matrices for pure and mixed states, and on the treatment of small numerical errors in the density matrices in the entropy calculations.

## Abbreviations

The following abbreviations are used in this manuscript:

GHZ	Greenberger–Horne–Zeilinger
NISQ	Noisy Intermediate-Scale Quantum
ECR	Echoed Cross-Resonance gate
SX	Error during the application of a single-qubit X gate
