# Supplementary material for: Entropy, Fidelity, and Entanglement During Digitized Adiabatic Quantum Computing to Form a Greenberger–Horne–Zeilinger (GHZ) State
Source: Entropy (Basel). 2025 Aug 23;27(9):891. doi: 10.3390/e27090891 (PMC12468138; doi:10.3390/e27090891)
Supplement: Supplementary file 1 [file entropy-27-00891-s001.zip › entropy-3769983-supplementary.pdf]

**Supplementary Material**

*Tables*

Table S1. The von Neumann entropy for quantum computers, simulators, and Python calculations
Table S2. Fidelity to the GHZ state for quantum computers, simulators, and Python calculations
Table S3. GHZ witness for quantum computers, simulators, and Python calculations
Table S4. Single-qubit purities for the two quantum computers, their simulators, and Python results
Table S5. Single-qubit von Neumann entropies for the systems in Table S4.
Table S6. Purities of the density matrices on ibm_sherbrooke, ibm_brisbane, and Python calculations
Table S7. GHZ witness in 4-, 8-, and 16-step adiabatic evolution processes
Table S8. Fidelity to the GHZ state in 4-, 8-, and 16-step adiabatic evolution processes
Table S9. Single-qubit entropies for qubit q[1] in 4-, 8-, and 16-step adiabatic evolution processes
Table S10. Single-qubit purities for qubit q[1] in 4-, 8-, and 16-step adiabatic evolution processes

*Figures*

Figure S1. Labeled city-scape plot
Figure S2. Fidelity to the GHZ state for quantum computers, simulators, and Python calculations
Figure S3. Single-qubit purities for the two quantum computers, their simulators, and Python results
Figure S4. Single-qubit von Neumann entropies for the systems in Figure S3
Figure S5. Purities of the density matrices on ibm_sherbrooke, ibm_brisbane, and Python calculations
Figure S6. GHZ witness in 4-, 8-, and 16-step adiabatic evolution processes
Figure S7. Fidelity to the GHZ state in 4-, 8-, and 16-step adiabatic evolution processes
Figure S8. Single-qubit entropies for qubit q[1] in 4-, 8-, and 16-step adiabatic evolution processes
Figure S9. Single-qubit purities for qubit q[1] in 4-, 8-, and 16-step adiabatic evolution processes

*Commentary*

Density matrices for pure and mixed states
Calculating the von Neumann entropy (treating small numerical errors)

**Table S1.** The von Neumann entropy S = – Tr(ρ ln ρ) obtained from the density matrices at each step of the adiabatic evolution on quantum computers, the quantum computer simulators, and in the Python calculations.

| | **Adiabatic evolution step** | | | | | | | | |
|---|---|---|---|---|---|---|---|---|---|
| **Entropy** | **0** | **1** | **2** | **3** | **4** | **5** | **6** | **7** | **8** |
| Python | 0.000000 | 0.000000 | 0.000000 | 0.000000 | 0.000000 | 0.000000 | 0.000000 | 0.000000 | 0.000000 |
| Fake Sherbrooke | 0.239472 | 0.275286 | 0.223740 | 0.267948 | 0.270805 | 0.319556 | 0.301065 | 0.324344 | 0.299241 |
| Fake Brisbane | 0.286581 | 0.334718 | 0.319695 | 0.351243 | 0.394690 | 0.412403 | 0.374151 | 0.419273 | 0.433255 |
| Fake Aachen | 0.385539 | 0.404203 | 0.400356 | 0.443371 | 0.480433 | 0.471739 | 0.505777 | 0.495125 | 0.530501 |
| Fake Perth | 0.402938 | 0.555229 | 0.674236 | 0.747614 | 0.906302 | 1.024690 | 1.166820 | 1.203170 | 1.251300 |
| ibm_sherbrooke | 0.211550 | 0.302067 | 0.396903 | 0.532685 | 0.603452 | 0.793417 | 0.912508 | 1.008060 | 1.209850 |
| ibm_brisbane | 0.336556 | 0.466812 | 0.684395 | 0.848229 | 1.274490 | 1.409570 | 1.549590 | 1.680280 | 1.891320 |

**Table S2.** Fidelity to the GHZ state during the adiabatic evolution process. Fidelity f = ⟨ GHZ | ρ | GHZ ⟩ obtained from the density matrix at each adiabatic evolution step on the quantum computers, the quantum simulators, and in the Python calculations

| | Adiabatic evolution step | | | | | | | | |
|---|---|---|---|---|---|---|---|---|---|
| **Fidelity** | **0** | **1** | **2** | **3** | **4** | **5** | **6** | **7** | **8** |
| Python | 0.250000 | 0.347580 | 0.392742 | 0.546764 | 0.732488 | 0.888456 | 0.986436 | 0.997990 | 0.997990 |
| Fake Sherbrooke | 0.240911 | 0.222930 | 0.295791 | 0.468248 | 0.634279 | 0.796597 | 0.906465 | 0.915243 | 0.920438 |
| Fake Brisbane | 0.228320 | 0.220534 | 0.301240 | 0.456401 | 0.622759 | 0.775747 | 0.896041 | 0.890527 | 0.891524 |
| Fake Aachen | 0.230172 | 0.213984 | 0.296535 | 0.441153 | 0.609097 | 0.771986 | 0.859533 | 0.874416 | 0.867094 |
| Fake Perth | 0.229414 | 0.218564 | 0.277218 | 0.402434 | 0.524157 | 0.621322 | 0.670247 | 0.642415 | 0.634522 |
| ibm_sherbrooke | 0.250870 | 0.239599 | 0.302269 | 0.437063 | 0.633173 | 0.728431 | 0.695170 | 0.618469 | 0.490356 |
| ibm_brisbane | 0.238963 | 0.218381 | 0.277841 | 0.413310 | 0.496216 | 0.562154 | 0.517965 | 0.458639 | 0.328328 |

**Table S3.** Results for the GHZ witness during the adiabatic evolution process. Values are listed for Tr[ρ $W_{GHZ}$] at each step, on the quantum computers, quantum simulators, and in the Python calculations.

| | Adiabatic evolution step | | | | | | | | |
|---|---|---|---|---|---|---|---|---|---|
| **GHZ Witness** | **0** | **1** | **2** | **3** | **4** | **5** | **6** | **7** | **8** |
| Python | 0.500000 | 0.402420 | 0.357258 | 0.203236 | 0.017512 | −0.138456 | −0.236436 | −0.247990 | −0.247990 |
| Fake Sherbrooke | 0.509089 | 0.527070 | 0.454209 | 0.281752 | 0.115721 | −0.046597 | −0.156465 | −0.165243 | −0.170438 |
| Fake Brisbane | 0.521680 | 0.529466 | 0.448760 | 0.293599 | 0.127241 | −0.025747 | −0.146041 | −0.140527 | −0.141524 |
| Fake Aachen | 0.519828 | 0.536016 | 0.453465 | 0.308847 | 0.140903 | −0.021986 | −0.109533 | −0.124416 | −0.117094 |
| Fake Perth | 0.520587 | 0.531436 | 0.472783 | 0.347567 | 0.225843 | 0.128678 | 0.079753 | 0.107585 | 0.115479 |
| ibm_sherbrooke | 0.499130 | 0.510401 | 0.447731 | 0.312937 | 0.116827 | 0.021569 | 0.054830 | 0.131531 | 0.259644 |
| ibm_brisbane | 0.511037 | 0.531619 | 0.472159 | 0.336690 | 0.253784 | 0.187846 | 0.232035 | 0.291361 | 0.421672 |

**Table S4**. Purities of the individual qubits, computed from the reduced density matrices at Tr($\rho_i^2$), where $\rho_i$ is the density matrix for qubit i. For a pure state, the purity is 1 and for a completely mixed state, the purity is 1/2.

| | Adiabatic Evolution Step | | | | | | | | |
|---|---|---|---|---|---|---|---|---|---|
| **Qubit q[0]** | **0** | **1** | **2** | **3** | **4** | **5** | **6** | **7** | **8** |
| Python | 1.000000 | 0.983904 | 0.968659 | 0.882135 | 0.744902 | 0.593200 | 0.508996 | 0.500681 | 0.500000 |
| Sherbrooke | 0.956012 | 0.911572 | 0.877849 | 0.775649 | 0.681757 | 0.555866 | 0.527961 | 0.526632 | 0.522181 |
| Fake Sherbrooke | 0.968847 | 0.916654 | 0.905427 | 0.798945 | 0.677016 | 0.581197 | 0.503164 | 0.500387 | 0.506693 |
| Brisbane | 0.983594 | 0.928255 | 0.883156 | 0.738627 | 0.577367 | 0.506060 | 0.503509 | 0.503328 | 0.501275 |
| Fake Brisbane | 0.957061 | 0.913346 | 0.903482 | 0.798994 | 0.680440 | 0.581540 | 0.503326 | 0.500520 | 0.507491 |
| **Qubit q[1]** | **0** | **1** | **2** | **3** | **4** | **5** | **6** | **7** | **8** |
| Python | 1.000000 | 0.969322 | 0.940262 | 0.817920 | 0.658804 | 0.554637 | 0.502054 | 0.501409 | 0.500309 |
| Sherbrooke | 0.965275 | 0.851537 | 0.829335 | 0.680966 | 0.571248 | 0.530809 | 0.540250 | 0.536505 | 0.531469 |
| Fake Sherbrooke | 0.944474 | 0.861364 | 0.851005 | 0.708074 | 0.545350 | 0.517144 | 0.522797 | 0.504274 | 0.500079 |
| Brisbane | 0.956368 | 0.850308 | 0.785718 | 0.632502 | 0.524527 | 0.500473 | 0.507524 | 0.504429 | 0.503337 |
| Fake Brisbane | 0.953839 | 0.868221 | 0.844239 | 0.695528 | 0.553987 | 0.516069 | 0.518839 | 0.505075 | 0.500186 |
| **Qubit q[2]** | **0** | **1** | **2** | **3** | **4** | **5** | **6** | **7** | **8** |
| Python | 1.000000 | 0.983904 | 0.968659 | 0.882135 | 0.744902 | 0.593200 | 0.508996 | 0.500681 | 0.500000 |
| Sherbrooke | 0.975266 | 0.901122 | 0.899206 | 0.778874 | 0.637420 | 0.549989 | 0.532152 | 0.533839 | 0.531082 |
| Fake Sherbrooke | 0.958695 | 0.911844 | 0.914039 | 0.801622 | 0.694583 | 0.580251 | 0.503511 | 0.501377 | 0.505616 |
| Brisbane | 0.895729 | 0.840353 | 0.763440 | 0.688155 | 0.576897 | 0.5041172 | 0.501673 | 0.500642 | 0.500618 |
| Fake Brisbane | 0.930781 | 0.882420 | 0.878561 | 0.775675 | 0.673572 | 0.577682 | 0.502666 | 0.500351 | 0.505648 |

**Table S5**. The von Neumann entropies of individual qubits during the adiabatic evolution process, computed as S = (-1) Tr ($\rho_i$ ln $\rho_i$), where $\rho_i$ is the reduced density matrix for qubit i. For a perfectly entangled qubit, S = ln 2 = 0.693147

| | Adiabatic Evolution Step | | | | | | | | |
|---|---|---|---|---|---|---|---|---|---|
| **Entropy of q[0]** | **0** | **1** | **2** | **3** | **4** | **5** | **6** | **7** | **8** |
| Python | 0.000000 | 0.047142 | 0.081721 | 0.234840 | 0.422830 | 0.596811 | 0.684124 | 0.692467 | 0.693147 |
| Sherbrooke | 0.107617 | 0.187666 | 0.241430 | 0.384009 | 0.498371 | 0.636192 | 0.664919 | 0.666274 | 0.670799 |
| Fake Sherbrooke | 0.081320 | 0.179133 | 0.197816 | 0.353531 | 0.503845 | 0.609596 | 0.689980 | 0.692760 | 0.686439 |
| Brisbane | 0.047898 | 0.159140 | 0.233260 | 0.430575 | 0.613650 | 0.687075 | 0.689634 | 0.690815 | 0.692406 |
| Fake Brisbane | 0.105539 | 0.184702 | 0.200994 | 0.353466 | 0.499894 | 0.609232 | 0.689817 | 0.692627 | 0.685638 |
| **Entropy of q[1]** | **0** | **1** | **2** | **3** | **4** | **5** | **6** | **7** | **8** |
| Python | 0.000000 | 0.080306 | 0.137577 | 0.327935 | 0.524641 | 0.637469 | 0.691091 | 0.691738 | 0.692838 |
| Sherbrooke | 0.088848 | 0.280603 | 0.312166 | 0.499286 | 0.620103 | 0.662014 | 0.652339 | 0.671014 | 0.673969 |
| Fake Sherbrooke | 0.129770 | 0.266216 | 0.281375 | 0.467509 | 0.647086 | 0.675904 | 0.670174 | 0.688867 | 0.693068 |
| Brisbane | 0.106911 | 0.282383 | 0.370956 | 0.554067 | 0.668415 | 0.692674 | 0.685604 | 0.688712 | 0.689807 |
| Fake Brisbane | 0.111883 | 0.256009 | 0.291116 | 0.482325 | 0.638144 | 0.676991 | 0.674188 | 0.688064 | 0.692961 |
| **Entropy of q[2]** | **0** | **1** | **2** | **3** | **4** | **5** | **6** | **7** | **8** |
| Python | 0.000000 | 0.047142 | 0.081721 | 0.234840 | 0.422830 | 0.596811 | 0.684124 | 0.692467 | 0.693147 |
| Sherbrooke | 0.067302 | 0.204826 | 0.207919 | 0.379848 | 0.548617 | 0.642290 | 0.660642 | 0.656184 | 0.661339 |
| Fake Sherbrooke | 0.102280 | 0.187212 | 0.183540 | 0.349963 | 0.483433 | 0.610598 | 0.689632 | 0.691769 | 0.687520 |
| Brisbane | 0.213495 | 0.296658 | 0.399605 | 0.490943 | 0.614147 | 0.688969 | 0.691473 | 0.692505 | 0.692529 |
| Fake Brisbane | 0.154683 | 0.234398 | 0.240339 | 0.383976 | 0.507805 | 0.613317 | 0.690479 | 0.692796 | 0.687488 |

**Table S6.** Purity of the density matrices in the adiabatic quantum computing process. Comparison of the purity $Tr(\rho^2)$ with the full density matrices, *versus* the result found with the real part of the density matrix alone, $Tr[(Re\,\rho)^2]$. Results from the quantum computers ibm_sherbrooke and ibm_brisbane, and Python calculations.

| ibm_sherbrooke | | | ibm_brisbane | | | Python | | |
|---|---|---|---|---|---|---|---|---|
| **Step** | **$Tr(\rho^2)$** | **$Tr[(Re\,\rho)^2]$** | **Step** | **$Tr(\rho^2)$** | **$Tr[(Re\,\rho)^2]$** | **Step** | **$Tr(\rho^2)$** | **$Tr[(Re\,\rho)^2]$** |
| 0 | 0.919792 | 0.919093 | 0 | 0.856765 | 0.855321 | 0 | 1.000000 | 1.000000 |
| 1 | 0.880014 | 0.807358 | 1 | 0.801495 | 0.745631 | 1 | 1.000000 | 0.998985 |
| 2 | 0.842645 | 0.756917 | 2 | 0.695944 | 0.631185 | 2 | 1.000000 | 0.998935 |
| 3 | 0.783374 | 0.627626 | 3 | 0.638063 | 0.520794 | 3 | 1.000000 | 0.988697 |
| 4 | 0.751847 | 0.659194 | 4 | 0.442001 | 0.397728 | 4 | 1.000000 | 0.982825 |
| 5 | 0.666559 | 0.607687 | 5 | 0.389196 | 0.356202 | 5 | 1.000000 | 0.974596 |
| 6 | 0.615232 | 0.546154 | 6 | 0.323062 | 0.312490 | 6 | 1.000000 | 0.991597 |
| 7 | 0.565865 | 0.457205 | 7 | 0.269989 | 0.256935 | 7 | 1.000000 | 0.996316 |
| 8 | 0.471861 | 0.348689 | 8 | 0.184950 | 0.178469 | 8 | 1.000000 | 0.996316 |

**Table S7.** The GHZ witness obtained from the density matrices in 4-step, 8-step and 16-step digitized adiabatic evolution processes, all from Python calculations. In order to facilitate comparisons at the same times, the 4-step results are listed for times 0, 2, 4, 6, 8; and the 16-step results are listed for times between 0 and 8 in increments of 0.5. The 8-step results are listed for times between 0 and 8 in increments of 1, as before.

**Adiabatic Evolution Step**

| **GHZ Witness** | **0** | **0.5** | **1** | **1.5** | **2** | **2.5** | **3** | **3.5** | |
|---|---|---|---|---|---|---|---|---|---|
| 4-step | 0.500000 | | | | 0.442021 | | | | |
| 8-step | 0.500000 | | 0.40242 | | 0.357258 | | 0.203236 | | |
| 16-step | 0.500000 | 0.472811 | 0.427764 | 0.399250 | 0.371311 | 0.319744 | 0.247653 | 0.165154 | |

| **GHZ Witness** | **4** | **4.5** | **5** | **5.5** | **6** | **6.5** | **7** | **7.5** | **8** |
|---|---|---|---|---|---|---|---|---|---|
| 4-step | -0.097601 | | | | -0.151299 | | | | -0.151299 |
| 8-step | 0.017512 | | -0.138456 | | -0.236436 | | -0.24799 | | -0.247990 |
| 16-step | 0.074746 | -0.017720 | -0.101319 | -0.170841 | -0.221284 | -0.244355 | -0.247934 | -0.248111 | -0.248111 |

**Table S8.** Fidelity to the GHZ state obtained from the density matrices in 4-step, 8-step and 16-step digitized adiabatic evolution processes, all from Python calculations. In order to facilitate comparisons at the same times, the 4-step results are listed for times 0, 2, 4, 6, 8; and the 16-step results are listed for times between 0 and 8 in increments of 0.5. The 8-step results are listed for times between 0 and 8 in increments of 1, as before.

**Adiabatic Evolution Step**

| **Fidelity** | **0** | **0.5** | **1** | **1.5** | **2** | **2.5** | **3** | **3.5** | |
|---|---|---|---|---|---|---|---|---|---|
| 4-step | 0.250000 | | | | 0.307979 | | | | |
| 8-step | 0.250000 | | 0.34758 | | 0.392742 | | 0.546764 | | |
| 16-step | 0.250000 | 0.277189 | 0.322236 | 0.350750 | 0.378689 | 0.430256 | 0.502347 | 0.584846 | |
| **Fidelity** | **4** | **4.5** | **5** | **5.5** | **6** | **6.5** | **7** | **7.5** | **8** |
| 4-step | 0.847601 | | | | 0.901299 | | | | 0.901299 |
| 8-step | 0.732488 | | 0.888456 | | 0.986436 | | 0.997990 | | 0.997990 |
| 16-step | 0.675254 | 0.767720 | 0.851319 | 0.920841 | 0.971284 | 0.994355 | 0.997934 | 0.998111 | 0.998111 |

**Table S9.** Single-qubit entropies for qubit q[1] obtained from the reduced density matrices in 4-step, 8-step and 16-step digitized adiabatic evolution processes, all from Python calculations. In order to facilitate comparisons at the same times, the 4-step results are listed for times 0, 2, 4, 6, 8; and the 16-step results are listed for times between 0 and 8 in increments of 0.5. The 8-step results are listed for times between 0 and 8 in increments of 1, as before.

**Adiabatic Evolution Step**

| **Entropy** | **0** | **0.5** | **1** | **1.5** | **2** | **2.5** | **3** | **3.5** | |
|---|---|---|---|---|---|---|---|---|---|
| 4-step | 0.000000 | | | | 0.024997 | | | | |
| 8-step | 0.000000 | | 0.080513 | | 0.137577 | | 0.327935 | | |
| 16-step | 0.000000 | 0.016130 | 0.054526 | 0.079086 | 0.114125 | 0.195910 | 0.286761 | 0.367972 | |
| **Entropy** | **4** | **4.5** | **5** | **5.5** | **6** | **6.5** | **7** | **7.5** | **8** |
| 4-step | 0.495383 | | | | 0.691337 | | | | 0.624193 |
| 8-step | 0.524641 | | 0.637469 | | 0.691091 | | 0.691738 | | 0.692838 |
| 16-step | 0.467531 | 0.566879 | 0.624850 | 0.658355 | 0.684777 | 0.691411 | 0.690949 | 0.693147 | 0.693074 |

The entropies of the three-qubit density matrices are very close to zero throughout the adiabatic evolution process in the Python calculations. With 4-steps, the entropy does not exceed $5.0 \cdot 10^{-14}$; with 8 steps, it does not exceed $1.5 \cdot 10^{-14}$, and with 16 steps, it does not exceed $8.5 \cdot 10^{-14}$.

**Table S10.** Single-qubit purities for qubit q[1] obtained from the reduced density matrices in 4-step, 8-step and 16-step digitized adiabatic evolution processes, all from Python calculations. In order to facilitate comparisons at the same times, the 4-step results are listed for times 0, 2, 4, 6, 8; and the 16-step results are listed for times between 0 and 8 in increments of 0.5. The 8-step results are listed for times between 0 and 8 in increments of 1, as before.

**Adiabatic Evolution Step**

| **Purity** | **0** | **0.5** | **1** | **1.5** | **2** | **2.5** | **3** | **3.5** | |
|---|---|---|---|---|---|---|---|---|---|
| 4-step | 1.000000 | | | | 0.992419 | | | | |
| 8-step | 1.000000 | | 0.969225 | | 0.940262 | | 0.81792 | | |
| 16-step | 1.000000 | 0.995456 | 0.980827 | 0.969891 | 0.952688 | 0.906589 | 0.847274 | 0.788001 | |
| **Purity** | **4** | **4.5** | **5** | **5.5** | **6** | **6.5** | **7** | **7.5** | **8** |
| 4-step | 0.684335 | | | | 0.501809 | | | | 0.567357 |
| 8-step | 0.658804 | | 0.554637 | | 0.502054 | | 0.501409 | | 0.500309 |
| 16-step | 0.708055 | 0.620857 | 0.566727 | 0.534387 | 0.508347 | 0.501735 | 0.502196 | 0.500000 | 0.500073 |

In the Python calculations, the purities of the three-qubit density matrices typically equal 1 to at least six significant figures. In fact, the purities are equal to 1 to fourteen significant figures in the 4-step and 16-step processes; exact purities for the 8-step process were not re-examined.

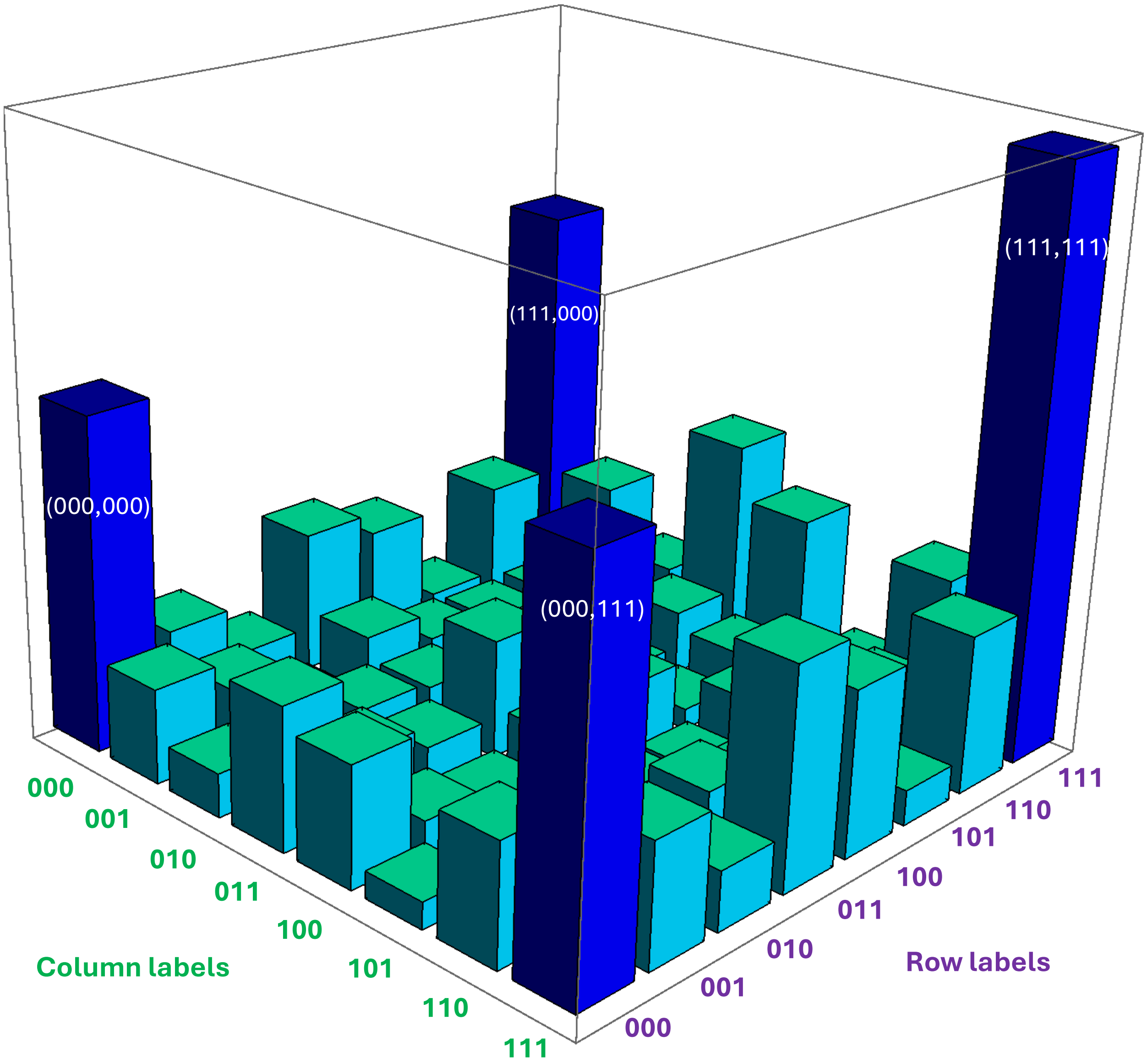


**Figure S1**. Labeled city-scape plot of the density matrix, with the state labels for the columns and rows indicated.

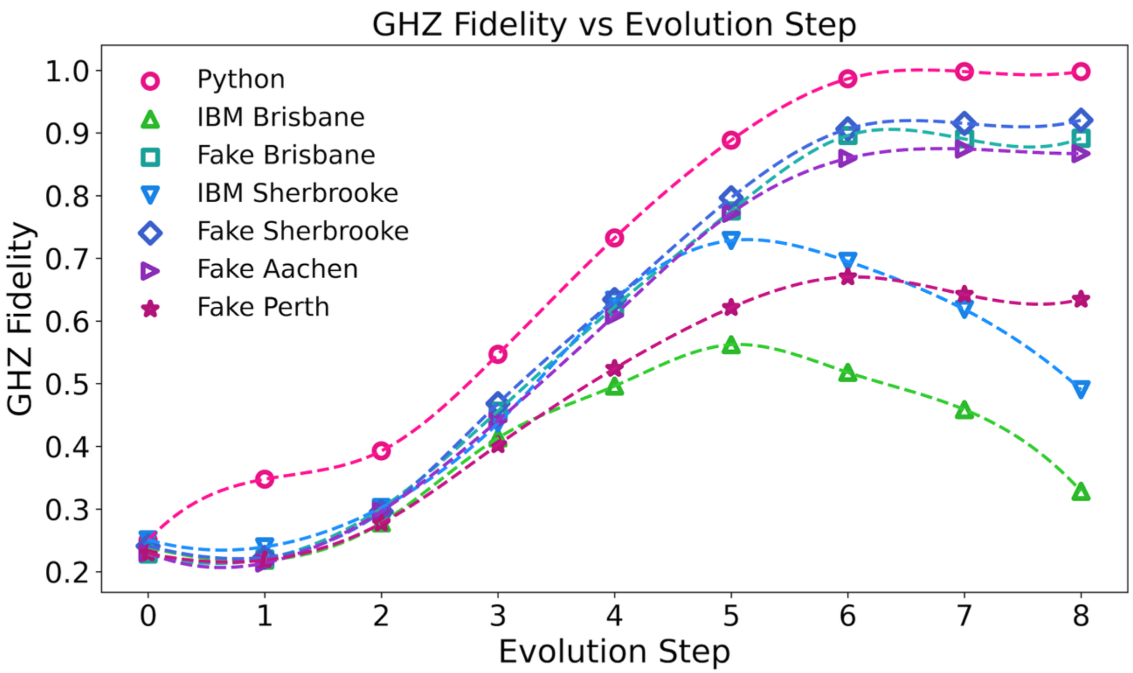


**Figure S2.** Fidelity to the GHZ state on the two quantum computers, the four quantum simulators, and in the Python calculations, as a function of the adiabatic evolution step.

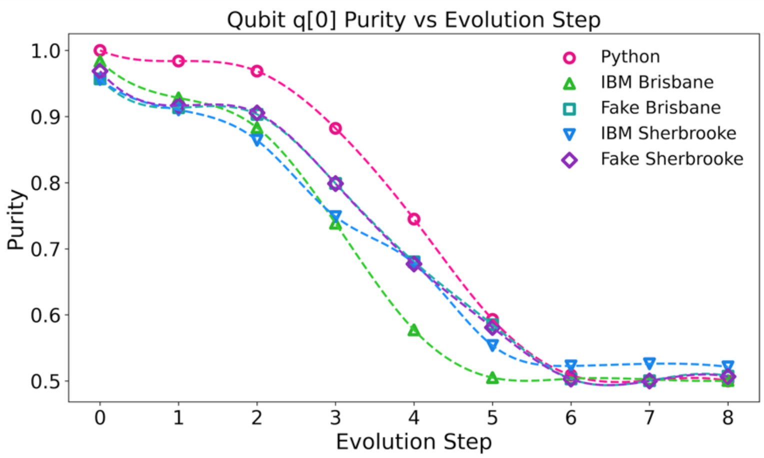


**(a)**

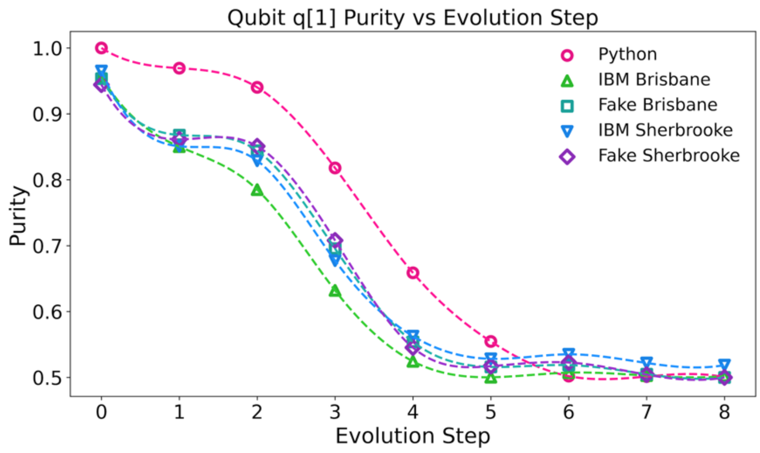


**(b)**

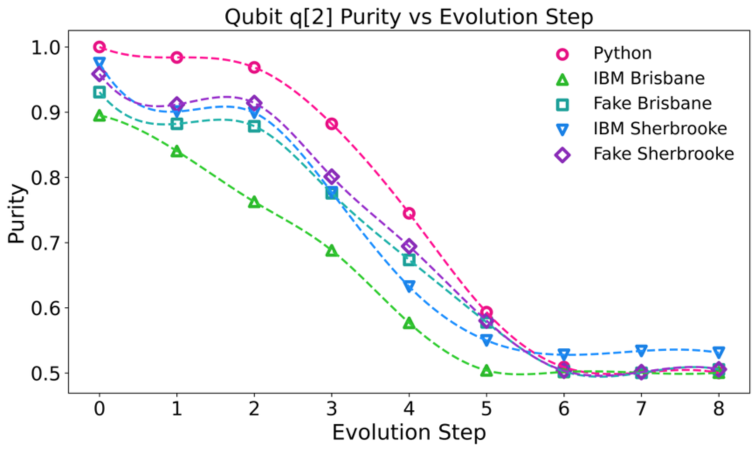


**(c)**

**Figure S3.** Purities of the individual qubits as a function of the step during the adiabatic evolution process, for the quantum computers ibm_sherbrooke and ibm_brisbane, their simulators, Fake Sherbrooke and Fake Brisbane, and the Python calculations: **(a)** Qubit q[0]; **(b)** Qubit q[1]; **(c)** Qubit q[2].

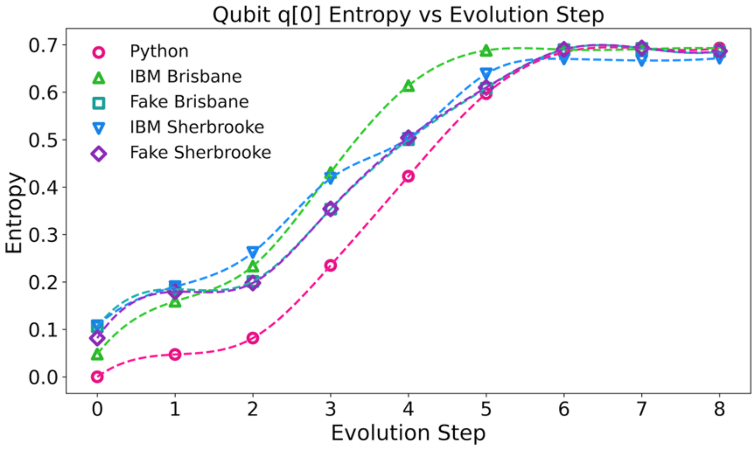


**(a)**

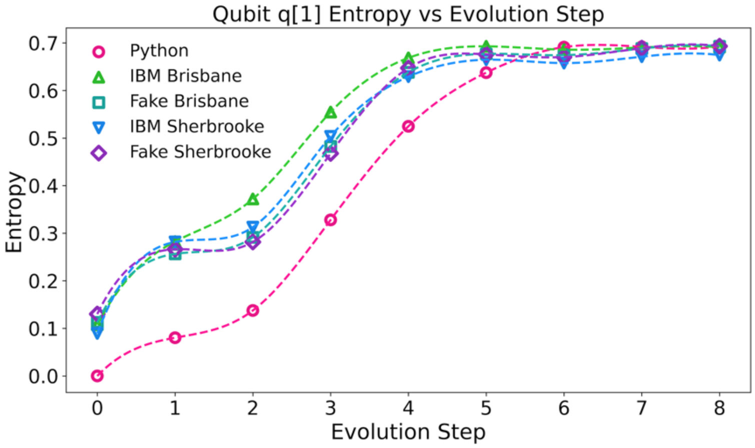


**(b)**

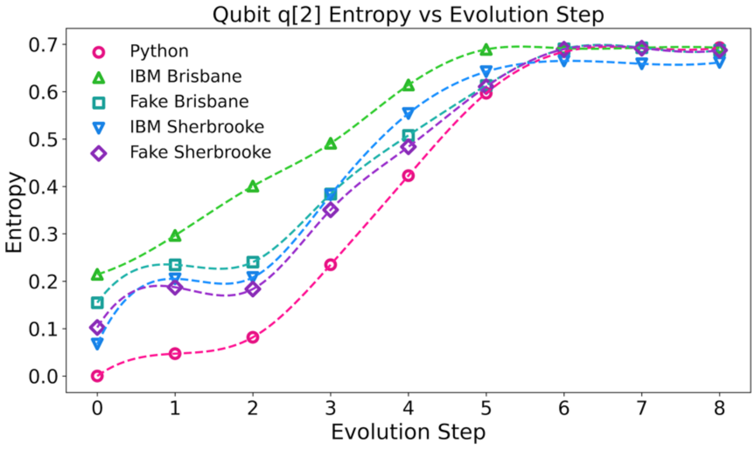


**(c)**

**Figure S4.** Entropies of the individual qubits as a function of the step during the adiabatic evolution process, for the quantum computers ibm_sherbrooke and ibm_brisbane, their simulators, Fake Sherbrooke and Fake Brisbane, and the Python calculations: **(a)** Qubit q[0]; **(b)** Qubit q[1]; **(c)** Qubit q[2].

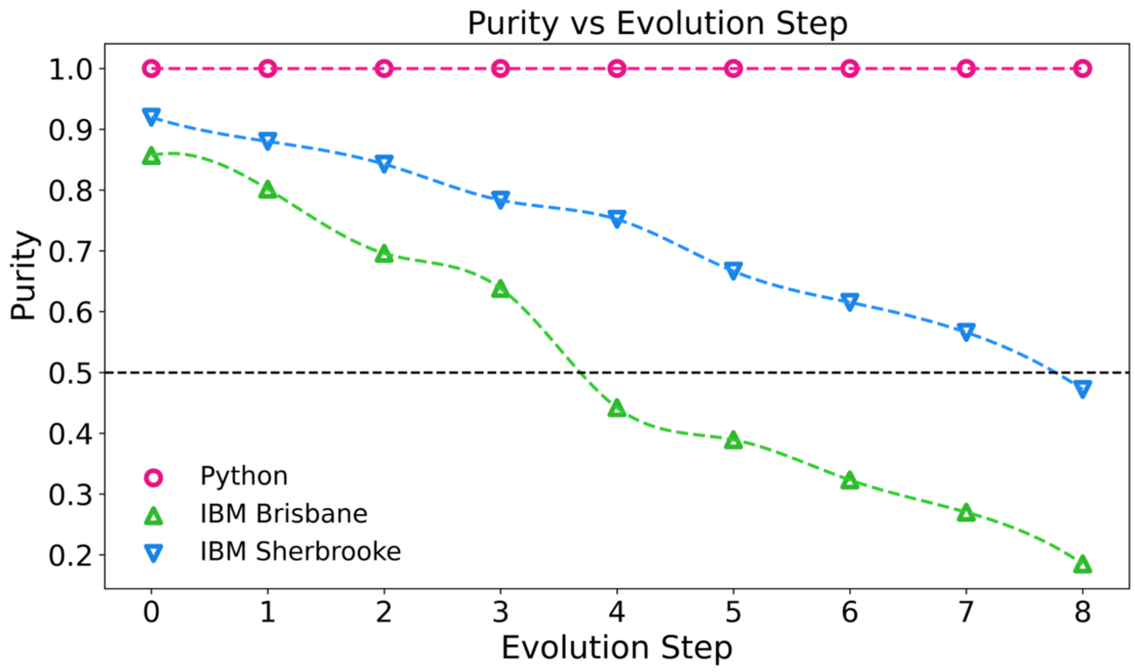


**Figure S5.** Purities of the density matrices on the two quantum computers, ibm_sherbrooke and ibm_brisbane, and in the Python calculations, as a function of the evolution step. For true adiabatic evolution, the purity would remain at 1.000000, as it does in the Python calculations.

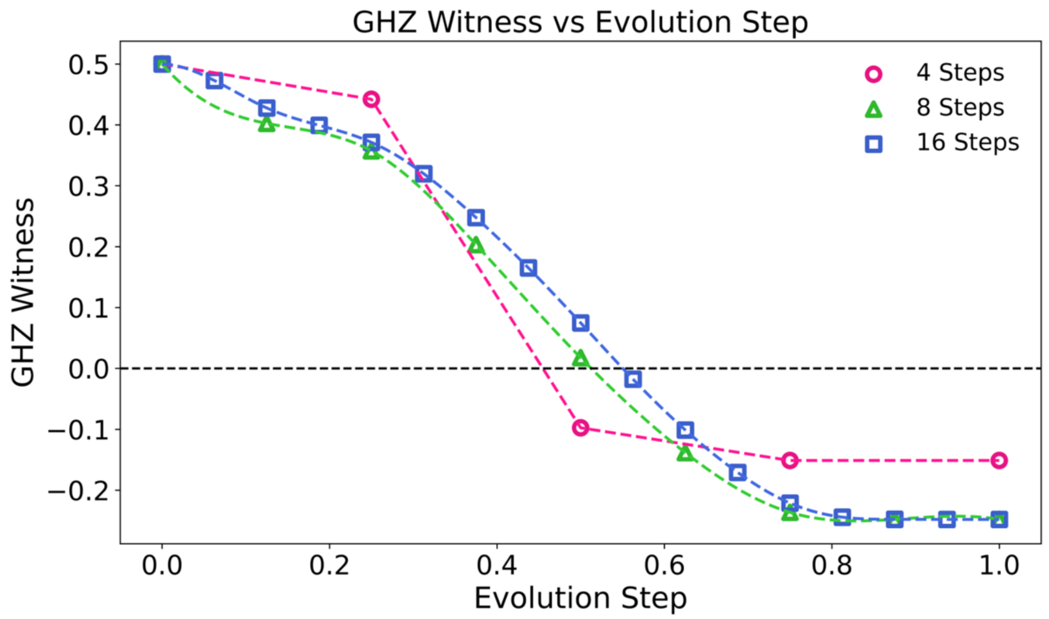


**Figure S6**. GHZ witness in 4-, 8-, and 16-step adiabatic evolution processes

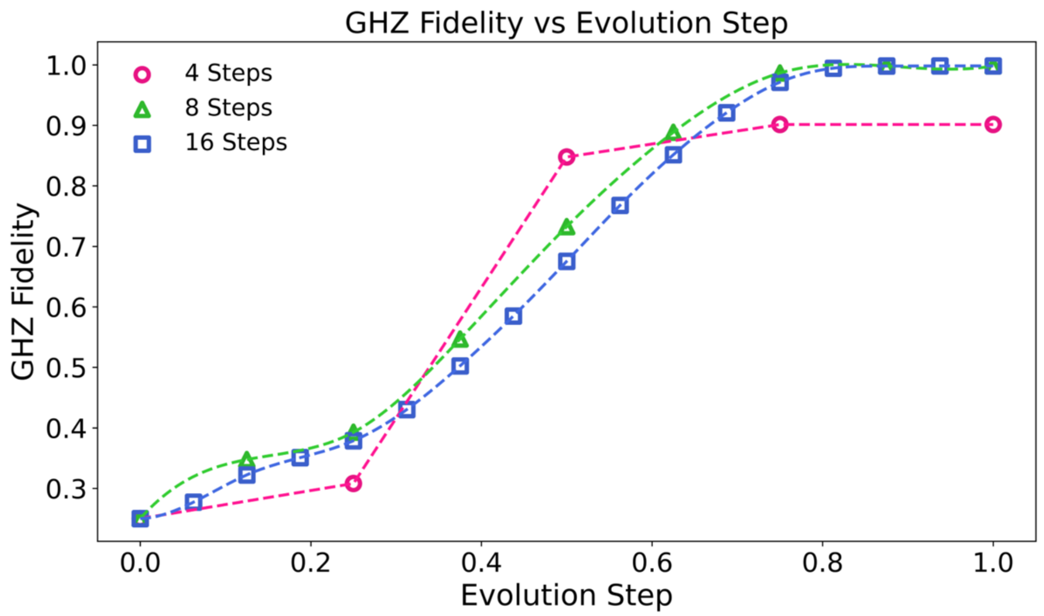


**Figure S7**. Fidelity to the GHZ state in 4-, 8-, and 16-step adiabatic evolution processes

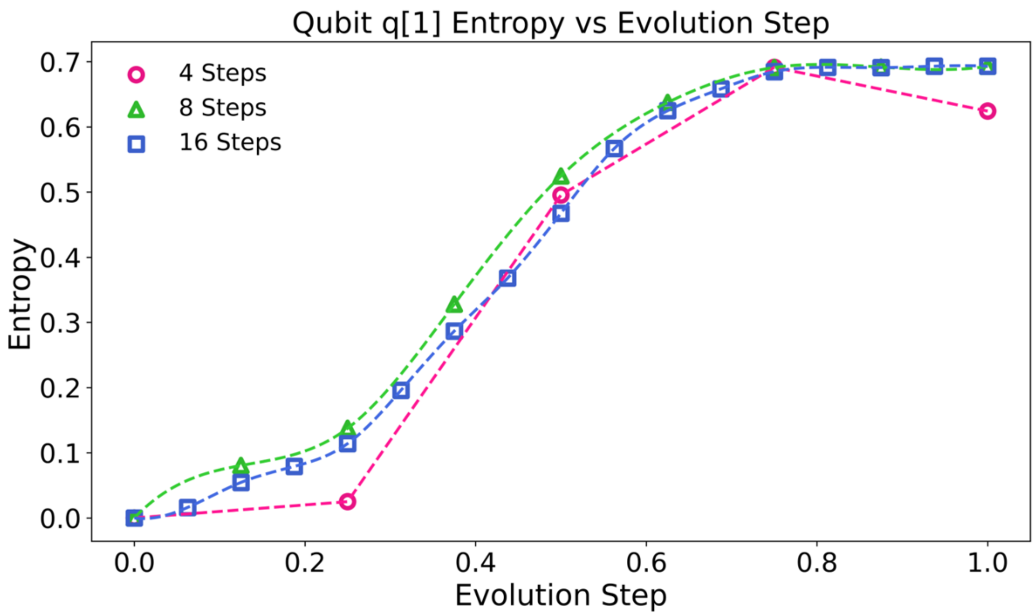


**Figure S8**. Entropies for qubit q[1] in 4-, 8-, and 16-step processes

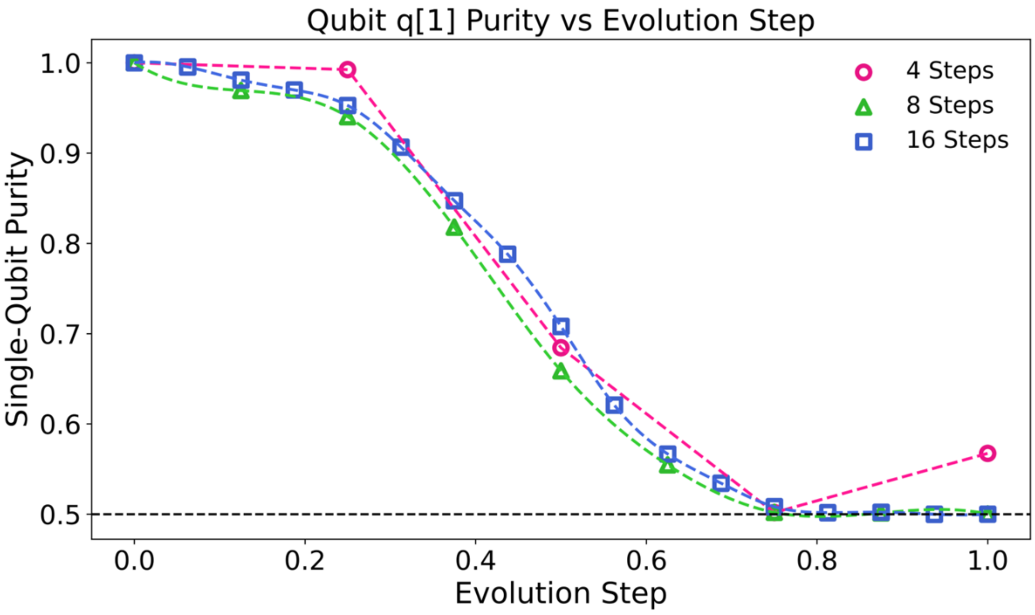


**Figure S9**. Purities for qubit q[1] in 4-, 8-, and 16-step processes

**Density matrix for a three-qubit pure state and for a three-qubit mixed state**

For the pure state $\Psi_s$, the density matrix operator is given by $\rho = | \Psi_s \rangle \langle \Psi_s |$

$| \Psi_s \rangle = c_{000} | 000 \rangle + c_{001} | 001 \rangle + c_{010} | 010 \rangle + c_{011} | 011 \rangle + c_{100} | 100 \rangle + c_{101} | 101 \rangle + c_{110} | 110 \rangle + c_{111} | 111 \rangle$

$\langle \Psi_s | = c_{000}^* \langle 000 | + c_{001}^* \langle 001 | + c_{010}^* \langle 010 | + c_{011}^* \langle 011 | + c_{100}^* \langle 100 | + c_{101}^* \langle 101 |$

$+ c_{110}^* \langle 110 | + c_{111}^* \langle 111 |$

In this case, we find the matrix element $\rho_{qr}$, where q and r are in the set {000, 001, 010, 011, 100, 101, 110, 111}, by applying the density matrix operator to $| r \rangle$ and then forming the scalar product with $\langle q |$

$\rho_{qr} = \langle q | \Psi_s \rangle \langle \Psi_s | r \rangle = c_q c_r^*$

A mixed state consists of an ensemble of pure states $| \Psi_k \rangle$, with probabilities $p_k$ that sum to 1. (Note that $p_k$ is a probability and not a probability amplitude). For a mixed state, the density matrix operator is given by $\rho = \Sigma_k p_k | \Psi_k \rangle \langle \Psi_k |$

We can express $| \Psi_k \rangle$ in the form $| \Psi_k \rangle = \Sigma_w c_{kw} | w \rangle$, where the states $| w \rangle$ are in the set { $| 000 \rangle$, $| 001 \rangle$, $| 010 \rangle$, $| 011 \rangle$, $| 000 \rangle$, $| 001 \rangle$, $| 010 \rangle$, $| 011 \rangle$ }. Then

$\langle \Psi_k | = \Sigma_w c_{kw}^* \langle w |$

In this case, to find $\rho_{qr}$ we note that within the term $| \Psi_k \rangle \langle \Psi_k |$ of the density matrix operator, we would have $\rho_{qr} = \langle q | \Psi_s \rangle \langle \Psi_s | r \rangle = c_q c_r^*$

Then the full density matrix for the mixed state has elements

$\rho_{qr} = \Sigma_k p_k c_{kq} c_{kr}^*$

where q and r are each an index for one of the basis in the set { $| 000 \rangle$, $| 001 \rangle$, $| 010 \rangle$, $| 011 \rangle$, $| 000 \rangle$, $| 001 \rangle$, $| 010 \rangle$, $| 011 \rangle$ }, and q and r may be identical.

References about density matrices, also given in the main text:


1. Fano, U. Description of states in quantum mechanics by density matrix and operator techniques. *Rev. Mod. Phys*. **1957**, *29*, 74-93.
2. Tolman, R. C., The Principles of Statistical Mechanics; Dover: Garden City, New York, USA, 2010.
3. Ungar, A. A. The density matrix for mixed state qubits and hyperbolic geometry. Quantum Info. & Comp. **2002**, *2*, 513-514.

**Details of the evaluation of the von Neumann entropy when numerical errors affect the density matrix**

The exact density matrices are Hermitian, so their eigenvalues are real. The eigenvalues of the density matrix are non-negative. Due to small numerical errors, the density matrices are not precisely equal to their complex conjugate transposes. As a result, we have occasionally found very small imaginary components on the order of $10^{-17}$ or $10^{-18}$ in the eigenvalues, and we have occasionally found very small negative eigenvalues on the order of $10^{-16}$ to $10^{-17}$. When both are kept in the calculations, we find a small imaginary component on the order of $10^{-17}$ in the trace of $\rho \ln \rho$. We have eliminated the imaginary components of the eigenvalues and the negative eigenvalues using the Mathematica instruction Chop [1], which replaces numbers that are very small by the integer 0. It is useful to note that the default tolerance for Chop is $10^{-10}$, many orders of magnitude larger than the imaginary or negative components that we have found in the eigenvalues. Then taking the logarithm directly, we find $\lim_{\varepsilon\to 0} \ln \varepsilon = -\infty$. However, since $\lim_{\varepsilon\to 0} \varepsilon \ln \varepsilon = 0$, the zeroes among the eigenvalues of the density matrix have no impact on the calculated von Neumann entropy. We have confirmed that the numerical result for the entropy obtained after using Chop agrees to at least six figures with the real part of the entropy, as calculated without the use of Chop.

1. Wolfram Research, Inc. Mathematica, Version 12.0.0.0. Champaign, IL (2024).
